# Quantification by Luminescence Tracking of Red Emissive Gold Nanoparticles in Cells

**DOI:** 10.1021/jacsau.0c00033

**Published:** 2021-01-19

**Authors:** Abiola N. Dosumu, Sunil Claire, Luke S. Watson, Patricia M. Girio, Shani A. M. Osborne, Zoe Pikramenou, Nikolas J. Hodges

**Affiliations:** ^†^School of Biosciences, ^‡^School of Chemistry, and ^§^Doctoral Training Centre in Physical Sciences for Health, The University of Birmingham, Edgbaston, Birmingham B15 2TT, United Kingdom

**Keywords:** gold nanoparticles, quantification, endosomal release, transition metal, autophagy

## Abstract

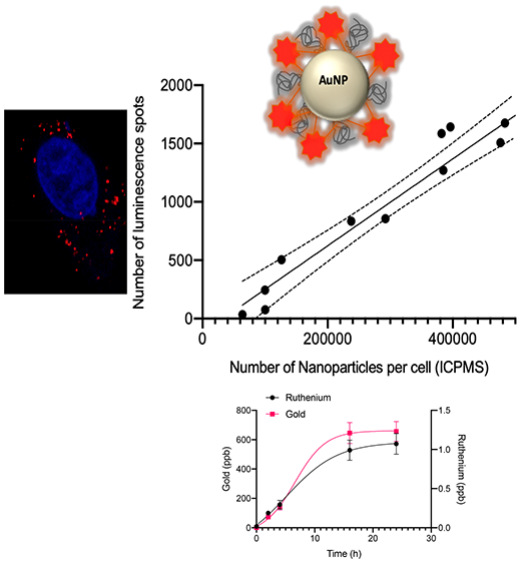

Optical microscopy techniques are ideal for live cell imaging for real-time nanoparticle tracking of nanoparticle localization. However, the quantification of nanoparticle uptake is usually evaluated by analytical methods that require cell isolation. Luminescent labeling of gold nanoparticles with transition metal probes yields particles with attractive photophysical properties, enabling cellular tracking using confocal and time-resolved microscopies. In the current study, gold nanoparticles coated with a red-luminescent ruthenium transition metal complex are used to quantify and track particle uptake and localization. Analysis of the red-luminescence signal from particles is used as a metric of cellular uptake, which correlates to total cellular gold and ruthenium content, independently measured and correlated by inductively coupled plasma mass spectrometry. Tracking of the luminescence signal provides evidence of direct diffusion of the nanoparticles across the cytoplasmic membrane with particles observed in the cytoplasm and mitochondria as nonclustered “free” nanoparticles. Electron microscopy and inhibition studies identified macropinocytosis of clusters of particles into endosomes as the major mechanism of uptake. Nanoparticles were tracked inside GFP-tagged cells by following the red-luminescence signal of the ruthenium complex. Tracking of the particles demonstrates their initial location in early endosomes and, later, in lysosomes and autophagosomes. Colocalization was quantified by calculating the Pearson’s correlation coefficient between red and green luminescence signals and confirmed by electron microscopy. Accumulation of particles in autophagosomes correlated with biochemical evidence of active autophagy, but there was no evidence of detachment of the luminescent label or breakup of the gold core. Instead, accumulation of particles in autophagosomes caused organelle swelling, breakdown of the surrounding membranes, and endosomal release of the nanoparticles into the cytoplasm. The phenomenon of endosomal release has important consequences for the toxicity, cellular targeting, and therapeutic future applications of gold nanoparticles.

## Introduction

Gold nanoparticles (AuNPs) have chemical and physical properties that make them unique multimodal probes for medical diagnostics and drug delivery.^1−4^ Attractive features include low toxicity and their ability to be functionalized to serve as a “scaffold” for attachment of luminescent probes. Luminescent labeling of AuNPs is a versatile approach for multimodal imaging introducing a luminescent readout signal based on the label while retaining the detection of gold based on its density with either optical or electron microscopy techniques. Luminescent nanoparticles can be monitored in live cells using conventional microscopy techniques, enabling time-resolved information to be collected to track AuNP uptake, fate, and drug delivery.^5^ We have previously used luminescent metals to decorate the surface of gold nanoparticles to produce luminescent nanoparticles with a large Stokes shift and high photostability^6−8^ and show cellular internalization in the absence of cytotoxicity.^9^ We have studied the design of the metal complexes for their distance from the gold particle to eliminate any quenching mechanisms from the gold. Ruthenium complexes with “long legs” for attachment to gold have been shown to be ideal probes, with enhancement of the ruthenium luminescence lifetime when attached to gold.^6^ Ruthenium and iridium complexes have long luminescence lifetimes, which enable multichannel lifetime imaging in cells with tracking both the gold signal in ps and the metal signal in ms.^10^ The optical signal of the probes is ideal to track the uptake mechanism and the fate of AuNPs inside cells, although it has not been utilized as a metric for quantification of AuNPs in cells, which usually relies on the isolation of cells and application of analytical techniques such as inductively coupled mass spectrometry (ICP-MS).

Uptake of gold nanoparticles into cells is typically by endocytic mechanisms, meaning that any cargo remains potentially trapped in the endosomal system where it may be subject to low pH and fusion with lysosomes, causing loss of functionality. Furthermore, nanoparticles entering cells by this pathway may also result in the activation of autophagy as an adaptive response to try and clear nanoparticles from the cell. Although potentially toxic, modulation of autophagy by nanoparticles has also been investigated as a way of sensitizing cancer cells to toxic payloads.^11^ To improve targeting to different parts of the cell, materials capable of escaping the endosomal system would be advantageous. Endosomal release of polymeric nanoparticles has been studied, and several biochemical mechanisms have been suggested including “proton-sponge” effects and fusion and damage of endosomal lipid membranes.^12,13^ Endosomal escape of chitosan-coated SPIONs^14^ and nanodiamonds^15^ have also been reported previously, but the issue of endosomal release of gold nanoparticles remains unexplored. Furthermore, the effect of the surface coating of the nanoparticles with luminescent labels may also influence their release and pathway into the cells.

In the current study, we investigate the uptake and time-resolved fate of luminescent ruthenium-labeled AuNPs by probing the ruthenium luminescence signal in live cells to demonstrate the pathway of the probe-coated AuNPs into the cells and use the red ruthenium signal for the quantification of AuNPs inside the cells. We chose a ruthenium complex, RuS12, as a label based on our previous studies that demonstrated it as an ideal probe with “long legs” leading to strongly luminescent particles with detection in the red region of the spectrum. We use the ruthenium signal to study the uptake of the AuNPs by cells and also electron microscopy to identify the mechanism of uptake. We introduce GFP-tagged fluorescent protein markers to correlate the ruthenium signal with the GFP signal at different times of uptake and study the fate of AuNPs inside autophagosomes.

Our approach introduces the ruthenium-modified particles as reliable tracking probes to quantify nanoparticle uptake with confocal microscopy methods, correlating them to analytical methods of whole cell population. The luminescent particles allow further insight to be gained into their intracellular fate and their limited endosomal release.

## Results and Discussion

### Quantification of Particle Uptake and Uptake in Early Endosomes

**Figure 1:**
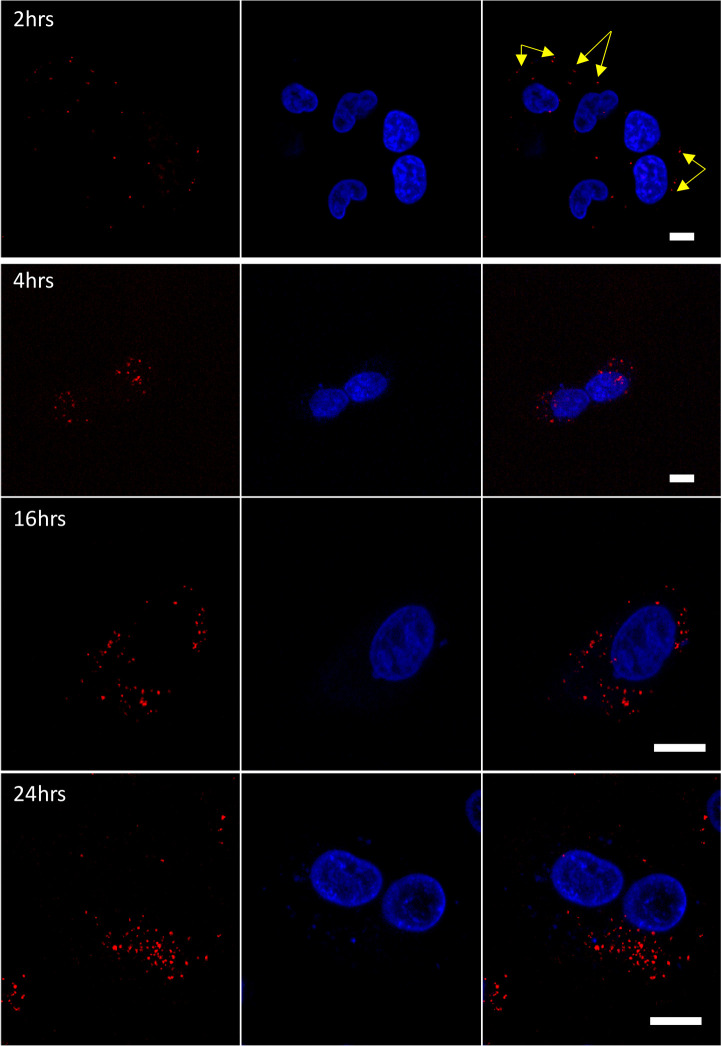
Live cell imaging of particle uptake inside of A549 cells. A549 cells were treated with 0.9 nM RuS12·AuNPs for 2, 4, 16, and 24 h. Red channel, RuS12·AuNP emission (λ_exc_ = 488 nm, λ_em_ 620–800 nm); blue channel, Hoechst emission (λ_exc_= 405 nm, λ_em_ = 410–455 nm). The yellow arrows indicate the presence of RuS12·AuNPs on the cytoplasmic membrane at the 2 h time point. The scale bar is 20 μm on all images.

Ruthenium-coated nanoparticles (RuS12·AuNPs) (Figure S1) were prepared and fully characterized for size and distribution by transmission electron microscopy (TEM) and dynamic light scattering (DLS) based on previous methods. The particles are spherical with a mean diameter of 15.5 ± 1.0 nm (95% confidence interval range is 14.9–16.1 nm as determined by TEM (Figure S1). Their spectroscopic properties agree with previous studies of luminescent ruthenium particles with characteristic ruthenium emission in the red, centered at 650 nm upon excitation at 488 nm (Figure S1). RuS12·AuNPs were used at a final nanoparticle concentration of 0.9 nM for all studies of cellular uptake and localization in A549 cells, which was determined to be noncytotoxic as assessed by the MTT assay at any of the time points investigated (Figure S2). Live cell uptake was monitored by confocal fluorescence microscopy (Figure 1) at 2, 4, 16, and 24 h of incubation and clearly demonstrated a time-dependent uptake of RuS12·AuNPs into cells. At early time points, the red ruthenium luminescence signal is located on or near the cytoplasmic membrane (yellow arrows), indicating association of particles or clusters of particles with the cytoplasmic membrane. Over time, the red signal is more widely distributed throughout the cell and apparent as punctuate perinuclear cytoplasmic staining.

**Figure 2:**
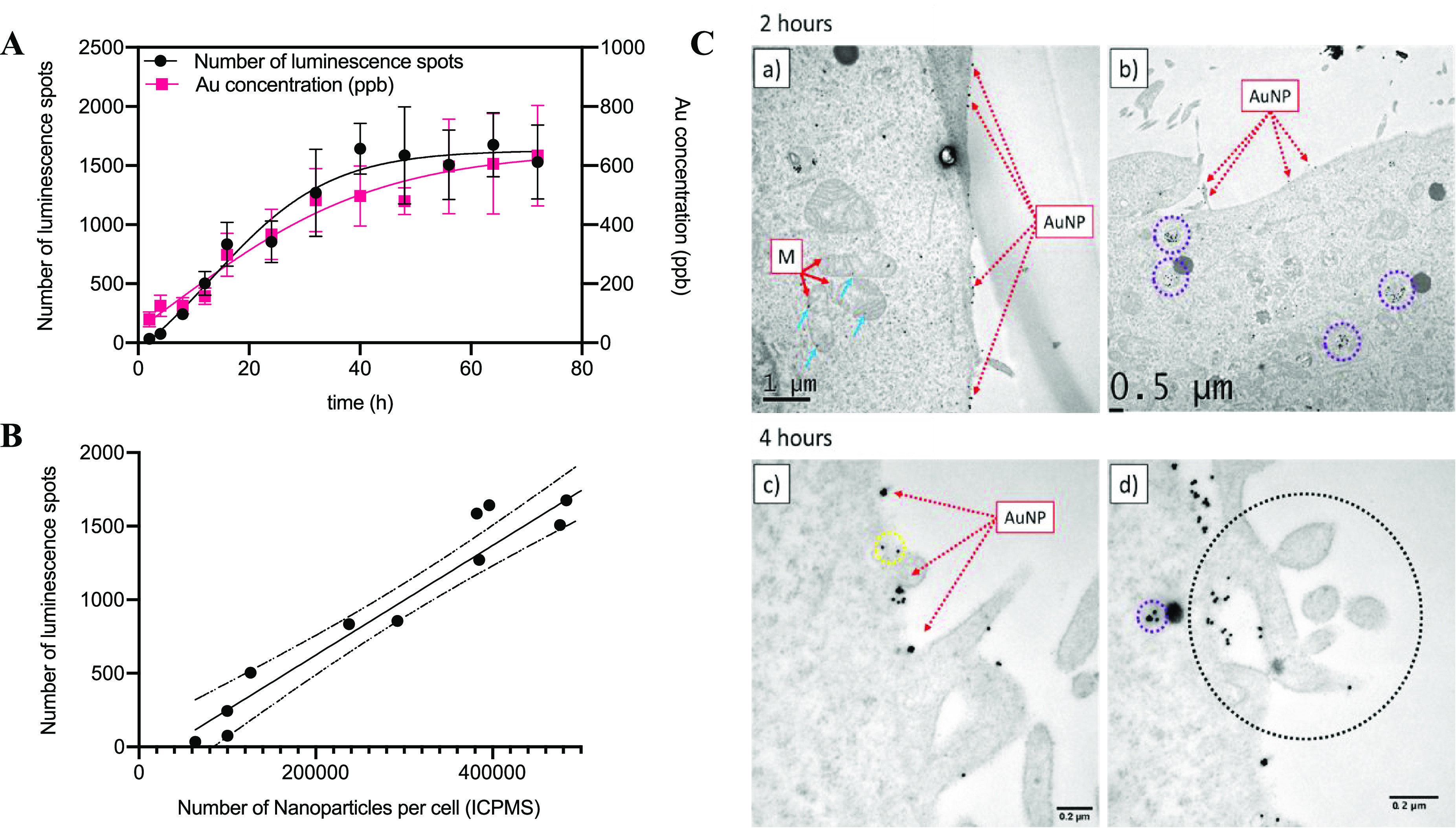
(A) Comparison of quantification of nanoparticle uptake into A549 cells using ICP-MS data (total cellular gold concentration) and confocal image analysis using the method described in Figure S3; the data generated represents cells analyzed from 10 random fields of view for each time point investigated. Fitted curves were generated with Prism v8 using a sigmoidal dose response model, *R*^2^ = 0.98 and 0.97, respectively. The predicted times taken to reach 50% maximal values were 25 and 19 h as assessed by ICP-MS and confocal analysis, respectively. (B) Linear-regression analysis between the number of RuS12·AuNPs per cell calculated from the ICP-MS data (*x* axis) and the number of luminescence spots in cells as quantified by confocal microscopy (*y* axis). There was a statistically significant linear correlation (*P* < 0.001, *F* = 129.9, *R*^2^ = 0.93). The dotted lines indicate 95% confidence boundaries for the line of best fit. (C) TEM images demonstrating uptake of RuS12·AuNPs in A549 cells following treatment with 0.9 nM RuS12·AuNPs for (a,b) 2 h and (c,d) 4 h. RuS12·AuNPs are clearly located on the cytoplasmic membrane (red arrows) as well as in vesicles in the vicinity of the cytoplasmic membrane (purple dotted circles) and mitochondria at these time points. In (a), blue arrows indicate RuS12·AuNPs in mitochondria (M), consistent with cellular uptake via translocation (yellow dotted circles), whereby RuS12·AuNPs can pass through the cell membrane, into the cytoplasm, and subsequently to mitochondria. In (d), the black dotted circle demonstrates evidence of membrane ruffling and protrusions around a cluster of nanoparticles consistent with cellular uptake via macropinocytosis.

Although confocal microscopy is widely used as a qualitative method to assess cellular uptake of AuNPs,^16^ ICP-MS is still considered the benchmark for the quantification of particles in cells assisted by TEM image analysis for localization. However, these techniques are time-consuming and of limited application when studying a large number of biological samples, for example, where detailed time-resolved information is required. Therefore, a robust method of particle quantification by fluorescence microscopy would be highly advantageous. Cellular quantification of nonluminescent AuNPs by quantifying weighting means particle scattering in cells exposed to 6 nm AuNPs has been reported previously,^17^ but this approach did not directly quantify particle number and gives no information about the particle distribution inside of cells. Here, we apply quantitative image analysis of particle number by probing the particle luminescence signal in threshold-confocal images of live cells (see Figure S3 for methodological details). The size of the nanoparticles limits their individual detection and tracking due to the diffraction limit of the conventional confocal microscopes. However, in this study, we have examined the luminescence signal of these bright ruthenium particles in the images of the focal planes to provide a correlation of the luminescence signal of the “luminescence spots” with the particle number of the whole cell quantified by ICP-MS. The “luminescence spots” were identified and quantified by ImageJ software using a binary image analysis. A calculated number of “luminescence spots” based on the red-luminescence signal at different time points is shown in Figure 2A. In a parallel experiment, we independently studied AuNP loading based on the detection of gold by ICP-MS. When the results obtained from the image quantification analysis were compared with the data obtained from ICP-MS quantification, linear-regression analysis showed a statistically significant (*P* < 0.001) linear correlation (*R*^2^ = 0.93) (Figure 2B), confirming agreement between the two quantification methods. Our data therefore indicates that in future studies, confocal image analysis of luminescence signal could be used to quantify luminescent AuNP uptake in cells.

Uptake of RuS12·AuNPs into live cells was further quantified using two additional end points: measurement of cellular ruthenium content by ICP-MS (Figure S4) and quantification of ruthenium fluorescence by flow cytometry (Figure S4). Both flow cytometry and ICP-MS confirmed the time-dependent uptake of particles observed using live cell imaging. Furthermore, there is a statistically significant linear correlation between these two parameters (*P* < 0.001, *F* = 56.6, *R*^2^ = 0.85), validating that ruthenium luminescence signal can be reliably used to quantify particles in cells. Furthermore, the ratio of ruthenium/gold by ICP-MS in the cells is found to be 1:260 (95% confidence range of 1:187 and 1:429) and the number of ruthenium complexes per nanoparticle was calculated by ICP-MS data to be 400 (95% confidence range of 243 to 556) based on a nanoparticle diameter of 15 nm as calculated for using the density of gold (Figure S1). Interestingly, in contrast to particles, there is very little cellular uptake of free ruthenium-probe RuS12 over the same time course as assessed by either end point, confirming that RuS12·AuNPs are able to efficiently deliver fluorescent ruthenium probe into cells. The concentration of the free probe used (0.6 μM) is an excess of RuS12 molecular probe based on the calculated number of complexes per nanoparticle. In addition, there was also good agreement between the cellular uptake of RuS12·AuNPs when cellular levels of gold and ruthenium levels were compared by ICP-MS. The correlation between cellular levels of gold and ruthenium was linear and statistically significant (*P* < 0.0001, *F* = 344.0, *R*^2^ = 0.97). (Figure S4).

Comparable time-dependence of nanoparticle localization was also obtained in fixed cells (Figure S5). A clear overlay between the red-luminescence and the gold reflectance signal confirming that there have been no intracellular decompositions of RuS12·AuNPs and release of free Ru probe at any of the time points investigated. We also confirmed the stability of RuS12·AuNPs up to 72 h in cell culture media in the presence and absence of glutathione (Figure S6). In addition, images of cells treated with free molecular label RuS12 (0.63 μM) for 4 h showed diffuse staining throughout the entire cytoplasm (Figure S7) that was completely different to that observed with RuS12·AuNPs, which remained localized in the vicinity of the cytoplasmic membrane at this time point. To further support the luminescence imaging results, transmission electron microscopy (TEM) was employed to provide high resolution localization of the particles. Consistent with the confocal microscopy images, TEM images acquired at the early time points of 2 and 4 h showed individual RuS12·AuNPs to be localized on and around the cytoplasmic membrane (Figure 2C). Analysis of 28 randomly selected particles from Figure 2C showed that RuS12·AuNPs in cells had a mean diameter of 15.0 ± 0.8 nm (95% confidence interval range 14.8–15.3 nm), which is consistent with the particle diameter of 15.5 ± 1.1 nm obtained in the cell-free particle preparation (Figure S1).

Small numbers of nonclustered particles both in the mitochondria and the cytoplasmic compartment of cells were also observed at these time points. When these are analyzed, false color mapping clearly demonstrates that the features interpreted as particles in the mitochondria are optically denser than the majority of “grainy” features in the cytoplasm of the same image. In contrast, they are similar in optical density to particles that are clearly located on the cell membrane (Figure S8). Furthermore, there is also evidence of nonclustered, “free” particles within the cytoplasm. In contrast, particles in mitochondria are not observable in our confocal images, where only larger accumulations of particles are resolvable as reported previously.^18^ Overall, our data suggest that although not a major mechanism of entry, some RuS12·AuNPs may enter cells directly by diffusion across the cytoplasmic membrane. Early studies appeared to have excluded this as a mechanism of nanoparticle uptake.^19^ However, more recent theoretical and experimental evidence^20−22^ supports the possibility of direct transfer of AuNPs across cell membranes. Our observation of RuS12·AuNPs free in the cytoplasm of cells is consistent with these findings. Furthermore, it is clear that this is not a major mode of cellular entry of RuS12·AuNPs. Interestingly, also clearly apparent was “ruffling” and projections of the cell membrane in regions of the cytoplasmic membrane containing particles (Figure 2C). These conformational changes to the cell membrane observed as fragments and protrusions are even more evident at 4 compared to 2 h (Figure 2C). Reshuffling and protrusion of the cell membrane indicates the involvement of macropinocytosis as a mechanism of cellular uptake. A similar TEM observation of plasma membrane protrusion and distortion on exposure to AuNPs has been observed in other cell lines^23−25^ as well as multiple types of Au nanoparticles,^26,27^ indicating that this mechanism of uptake is insensitive to particle surface coating.

**Figure 3:**
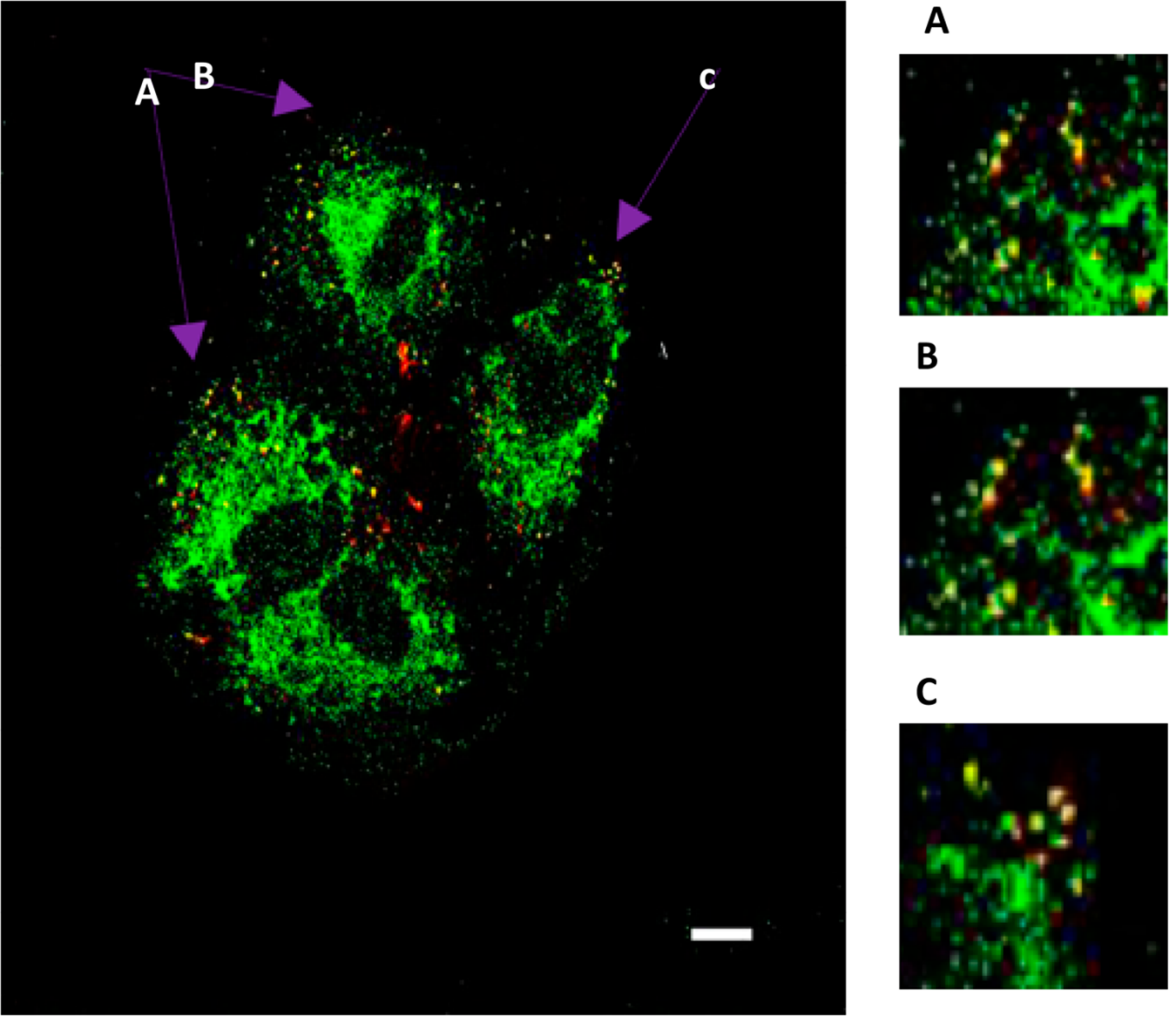
Confocal microscopy demonstrating the localization of RuS12·AuNPs in Rab4-GFP-positive early endosomes. Representative image of A549 transiently transfected with GFP-Rab4 and treated with 0.9 nM RuS12·AuNPs for 4 h. Red channel, RuS12·AuNP emission (λ_exc_ = 488 nm, λ_em_ 620–800 nm) and green channel, GFP emission (λ_exc_ = 488 nm, λ_em_ = 502 nm). Zoomed regions A–C clearly show areas of colocalization between the red and green channels indicating the presence of RuS12·AuNPs in Rab4-positive (early) endosomes. Scale bar = 10 μm.

Macropinocytosis is usually associated with uptake of larger particles in the order of 200 nm, which suggests that clusters of RuS12·AuNPs that had accumulated on the cytoplasmic membrane rather than individual particles are absorbed by this pathway. This is consistent with our results of membrane associated luminescence by confocal microscopy, where the resolution is not sufficient to visualize individual particles in the vicinity of the cell membrane. The appearance of smaller invaginations of plasma membrane as well as evidence of particles in vesicles bound by single membranes (Figure 2C) also support involvement of clathrin-mediated endocytosis as another mechanism of uptake of RuS12·AuNPs. To investigate the relative importance of these two pathways further, cells were pretreated with 50 μM chlorpromazine (CPZ), an inhibitor of receptor-mediated endocytosis. CPZ had no statistically significant effect on the uptake of RuS12·AuNPs as quantified by flow cytometry (Figure S9) confirming that macropinocyotosis is the major endosomal mechanism of cellular uptake of RuS12·AuNPs. As a positive control, uptake of FITC-conjugated transferrin was inhibited by 57% by incubation with CPZ (Figure S9). To confirm that particles were trafficked into the endosomal compartment, cells were colabeled with GFP-tagged Rab4, a protein marker of early endosomes. Colocalization of the red-luminescent signal from nanoparticles and Rab4-GFP was only observed at early time points (4 h) and only in the vicinity of the cell membrane (Figure 3). There was no colocalization between Rab4 and RuS12·AuNPs at any of the later time points investigated, suggesting that once inside early endosomes, particles are rapidly sorted to other vesicles and trafficked to other endosomal compartments of the cell.

To further study possible mitochondrial localization of RuS12·AuNPs, cells were colabeled with MitoTracker Green, but there was only limited evidence of any colocalization of red-luminescence signal from RuS12·AuNPs with mitochondria (Figure S10), suggesting that although individual particles not resolvable by confocal microscopy may be associated with the mitochondrial compartment, the majority of particles are present as cellular nanoparticle clusters that are restricted to endosomal compartments of the cell. There was also some limited evidence of luminescence signal within the nucleus as well, suggesting that this compartment is also potentially accessible to RuS12·AuNPs. (Figures S10 and S11). This and the limited evidence of mitochondrial localization of RuS12·AuNPs is consistent with previous findings from our group investigating the intracellular fate of similar iridium-coated AuNPs, which also were largely restricted to endosomal compartments but had limited access to other compartments of the cell as evidenced both by imaging and ICP-MS.^10^

### Trafficking of RuS12·AuNPs into the Lysosomal Pathway

**Figure 4:**
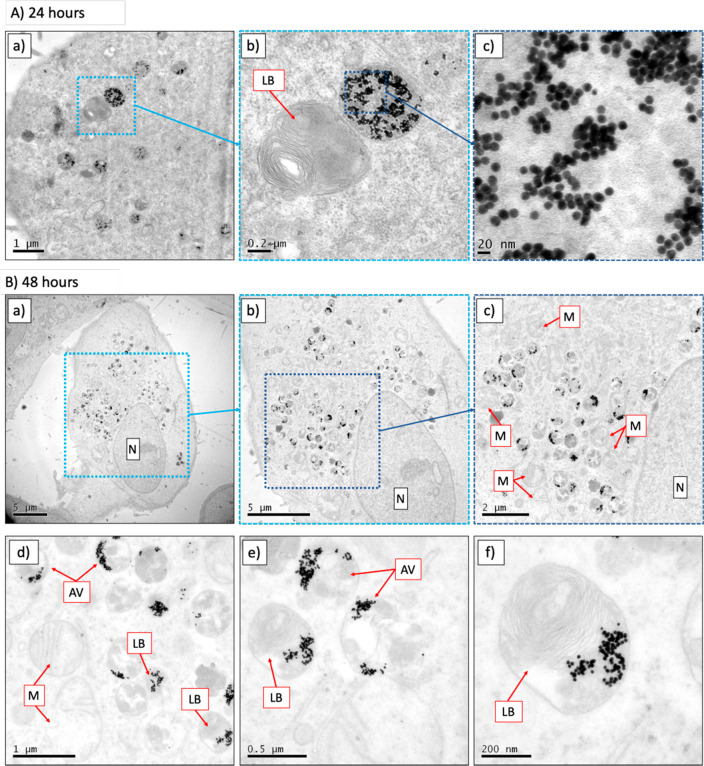
TEM images demonstrating localization of RuS12·AuNPs in multiple vesicular compartments of the cell after A549 cells were treated with 0.9 nM RuS12·AuNPs. (A) 24 h, zoomed images (b,c) show localization of particles in a possible lysosome and demonstrate that RuS12·AuNPs are still spherically monodispersed with no evidence of any aggregations or breakdown. (B) 48 h, (a–c) particles are found to be localized in vesicles. There was no evidence of any colocalization with the mitochondria (M) or cell nucleus (N) in (a–c). Images (d–f) show evidence of localization of particles in multiple vesicles including lamella bodies (LB) and autophagic vacuoles (AV).

Once inside the endosomal compartment of cells, there are three major trafficking pathways: (1) the degrative pathway involving fusion with lysosomes, (2) transfer to the “trans-Golgi” network with membrane recycling, and (3) transfer to perinuclear endosomes and subsequent membrane recycling.^28^ To investigate a possible role of the trans-Golgi network in intercellular trafficking of RuS12·AuNPs, cells were colabeled with GOLGI ID, but there was no evidence for any luminescence signal from RuS12·AuNPs within this compartment of the cell at 24 h (Figure S11). Lack of association with the Golgi strongly suggests that the intracellular fate of RuS12·AuNPs is the degradative pathway and fusion with lysosomes. In support of this, TEM images of cells treated with RuS12·AuNPs for 24 and 48 h clearly showed that particles remained largely localized inside of vesicles with, as discussed above, only evidence of small numbers of free particles in the cytoplasm or in the mitochondria of cells. The morphology of the structures containing RuS12·AuNPs is consistent with multivesicular bodies (MVBs), a specialized form of late endosomes, consisting of membrane-bound intraluminal vesicles^29^ as well as autophagic vesicles (Figure 4). Interestingly, at all the time points investigated, the RuS12·AuNPs remained as monodispersed particles with no evidence of intracellular aggregation, demonstrating their stability and resistance to degradation within the intracellular environment (Figure 4).

**Figure 5:**
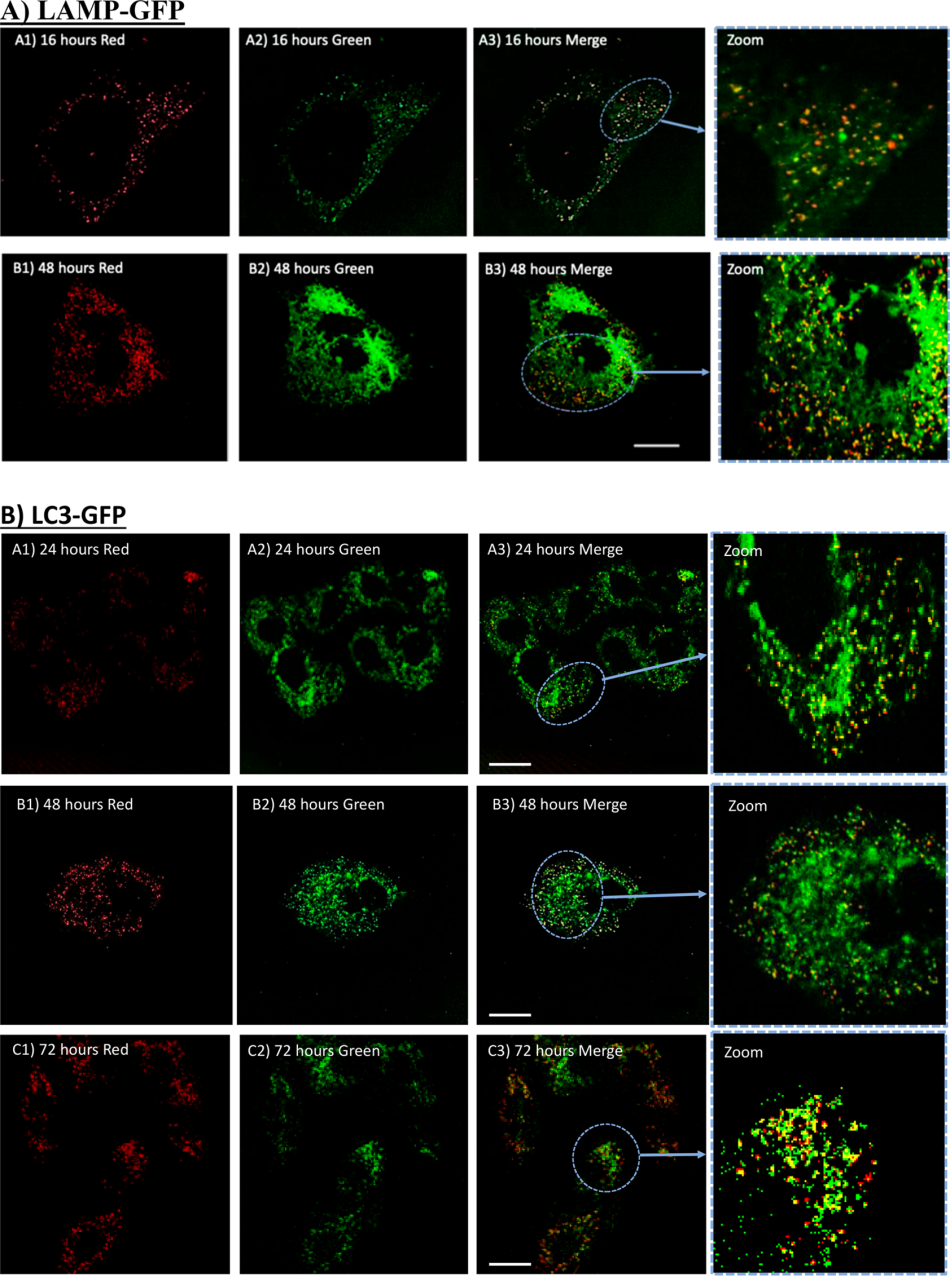
(A) Confocal microscopy demonstrating the localization of RuS12·AuNPs in LAMP-GFP-positive lysosomes. Representative image of A549 transiently transfected with GFP-LAMP and treated with 0.9 nM RuS12·AuNPs for 16 and 48 h. Red channel, RuS12·AuNP emission (λ_exc_ = 488 nm, λ_em_ = 620–800 nm) and green channel, GFP emission (λ_exc_ = 488 nm, λ_em_ = 502 nm). Zoomed regions clearly show areas of colocalization between the red and green channels indicating the time-dependent colocalization of RuS12·AuNPs in LAMP-GFP-positive lysosomes. Scale bar = 20 μm. (B) Time-dependent colocalization of RuS12·AuNPs in LC3-GFP-positive autophagosomes shown by confocal microscopy. Representative image of A549 transiently transfected with GFP-LC3 and treated with 0.9 nM RuS12·AuNPs for 24, 48, and 72 h. Red channel, RuS12·AuNP emission (λ_exc_ = 488 nm, λ_em_ = 620–800 nm) and green channel, GFP emission (λ_exc_ = 488 nm, λ_em_ = 502 nm). Zoomed regions clearly show areas of colocalization between the red and green channels. The scale bars represent 20 μm.The correlation of the two signals is shown in Figure 6A.

**Figure 6:**
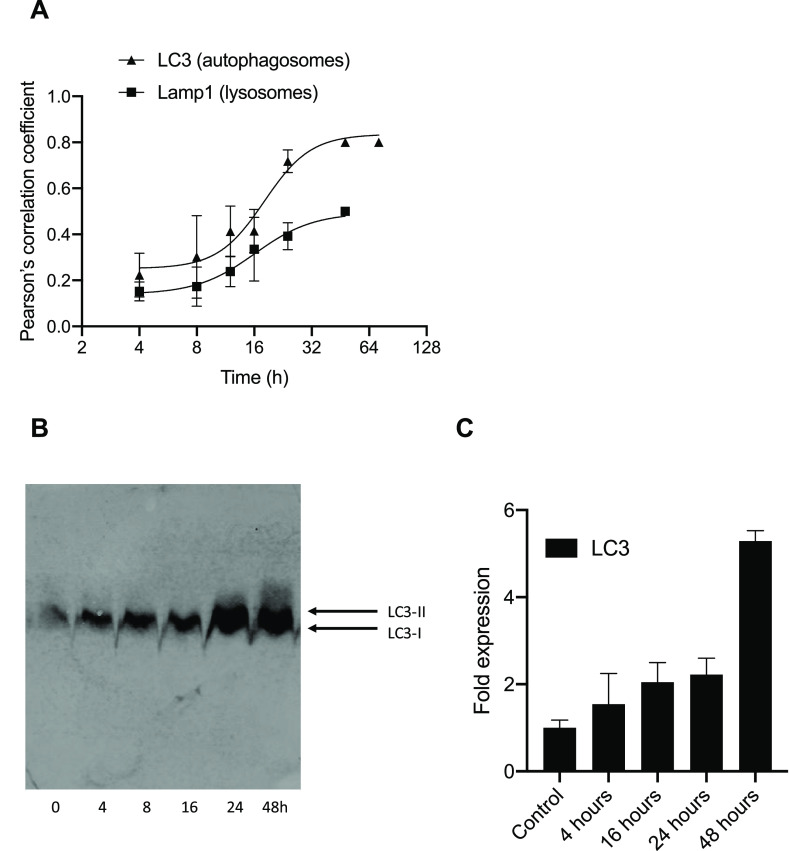
(A) Time-dependent increase in Pearson’s correlation coefficient (PCC) values for RuS12·AuNPs colocalized with the lysosomal (Lamp1) and autophagosomal (LC3) compartments of A549 cells. Data were fitted to a sigmoidal dose–response model in Prism V8, *R*^2^ = 0.50 and 0.49, respectively. For each time point between 0–24 h, PCC correlations were calculated from four representative cells. For the 48 and 72 h time points, the data represents that obtained from a single representative cell. (B) Biochemical evidence of activation of autophagy in RuS12·AuNP treated cells. A549 cells were treated with 0.9 nM RuS12·AuNPs for 2, 4, 8, 16, 24, and 48 h, and induction of LC3 protein was detected by Western blotting. (C) Induction of LC3 as assessed by qPCR of A549 cells exposed to 0.9 nM RuS12·AuNPs for 0, 4, 16, 24, 48, and 72 h. A549 cells were treated with 0.9 nM RuS12·AuNPs for 0, 4, 16, and 24 h. At the end of each time point, RNA was extracted and reverse transcribed to cDNA. cDNA was used for RT-PCR with TaqMan gene expression. The experiment is a biological triplicate for each time point with three technical replicates.

Lamella bodies are specialized membrane-bound organelles that are in dynamic equilibrium via fusion with other vesicles including MVBs and lysosomes.^30^ Localization of RuS12·AuNPs in lamella bodies was also clearly observed by TEM (Figure 4). A major fate of MVBs is fusion with lysosomes; to confirm the presence of RuS12·AuNPs in the lysosomal compartment, cells were transfected with GFP-tagged LAMP1, a specific marker of lysosomes. Colocalization of this luminescence signal from RuS12·AuNPs with GFP-LAMP1 over 72 h showed a time-dependent increase from 16–48 h (Figure 5A), which was quantified by determining the Pearson correlation coefficient (PCC) between the ruthenium fluorescence and GFP signal, which increased in a time-dependent way from 0.33 at 16 h to 0.39 after 48 h and 0.50 at 48 h (Figure 6A). Previous studies with different types of AuNPs including citrate capped, cationic, and polyethylene-glycol-coated materials have reported a similar accumulation within the lysosomal compartment of cells, suggesting that this is a canonical cellular fate for gold nanomaterials independent of size and surface coating.^27,31,32^

### Autophagy and Endosomal Release at Late Time Points Induced by RuS12·AuNPs

Autophagosomes are double membrane vesicles that are formed around the intracellular substrate including regions of the cytoplasm and organelles including mitochondria. They target their contents for lysosomal degradation by lysosomal hydrolases.^33^ Autophagosomes are transient and highly inducible under various conditions including cellular stress. They are believed to be assembled *de novo* in the cytoplasm,^34^ and LC3 is a protein that plays a central role in autophagosome function, specifically their maturation. To investigate the role of autophagy in the intracellular fate of RuS12·AuNPs, cells were labeled with GFP-tagged LC3 protein. In untreated control cells, LC3-GFP staining was predominantly diffusely distributed throughout the cytoplasm with only limited evidence of discrete foci of staining indicative of active autophagy (Figure S12). In contrast, in cells treated with RuS12·AuNPs, a change to a more localized punctuate staining pattern was observed in a time-dependent manner from 24 to 72 h, demonstrating the formation of active autophagosomal vesicles inside cells treated with RuS12·AuNPs. Clear colocalization between LC3-GFP and red-luminescent nanoparticle signals confirmed the presence of RuS12·AuNPs in LC3-positive autophagosomes (Figure 5B).

Association of RuS12·AuNPs with autophagosomes was also evaluated quantitatively by calculating the PCC values of the red (particle) and green (GFP) fluorescence channels, which were 0.41, 0.71, 0.8, and 0.8, for 16, 24, 48, and 72 h, respectively, with evidence of a plateau after 48 h of treatment (Figure 6A). Western blotting further confirmed that RuS12·AuNPs induce autophagy with LC3 induction observed in a time-dependent manner and with clear evidence of both the LC3-I and LC3-II bands at approximately 18 and 16 kDa, respectively, confirming the presence of active autophagosomes at 24 and 48 h time points (Figure 6B). Induction of LC3 was also confirmed at the mRNA level by qPCR (Figure 6C).

Previous studies have shown that multiple classes of metal-core nanoparticles accumulate in autophagosomes and modulate autophagy.^35^ Although activation of autophagy by nanoparticles could be viewed as a toxic cellular response, it also has therapeutic potential, and targeting the autophagosomal pathway with AuNPs could potentially be used to treat a number of metabolic diseases as well as cancer.^35−41^

In the current study, there was clear evidence of RuS12·AuNPs within the lysosomal and autophagosomal compartments of cells. Interestingly,^37^ it was observed that uncoated AuNPs caused activation of autophagy in a manner that was size-dependent, with larger nanoparticles (50 nm) being more potent activators than smaller (10 and 25 nm) particles. The mechanism of action appeared to be the inhibition of turnover of endogenous autophagosomes rather than direct activation of autophagy. Consistent with this, in the current study, we did not detect any evidence of oxidative stress as quantified by oxidation of the redox sensitive probe dichlorofluoroscin diactete or heat shock protein 70 (HSP70) induction (Figure S13), which are normally associated with direct activation of autophagy in cells. Likewise, previous studies by Hauck et al.^42^ also showed no changes in HSP70 after exposure to AuNPs. Furthermore, we observed an increase in the lysosomal pH of cells treated with RuS12·AuNPs (Figure S14) that is consistent with previous reports of alkalinization of components of the endosomal system by AuNPs and accumulation of autophagy related vesicles.^37^ Despite the progress made in elucidating the mechanism of how AuNPs activate autophagy, the long-term fate of particles in autophagosomes is largely unknown. Although previous studies have used photothermal and laser activation methods to trigger endosomal release of AuNPs, for example, to improve transfection efficiency of plasmid DNA,^43^ in these studies, there was no evidence of AuNPs free in the cytoplasm prior to treatment,^44^ and it was unclear whether the reported endosomal release was associated with cellular toxicity.

**Figure 7:**
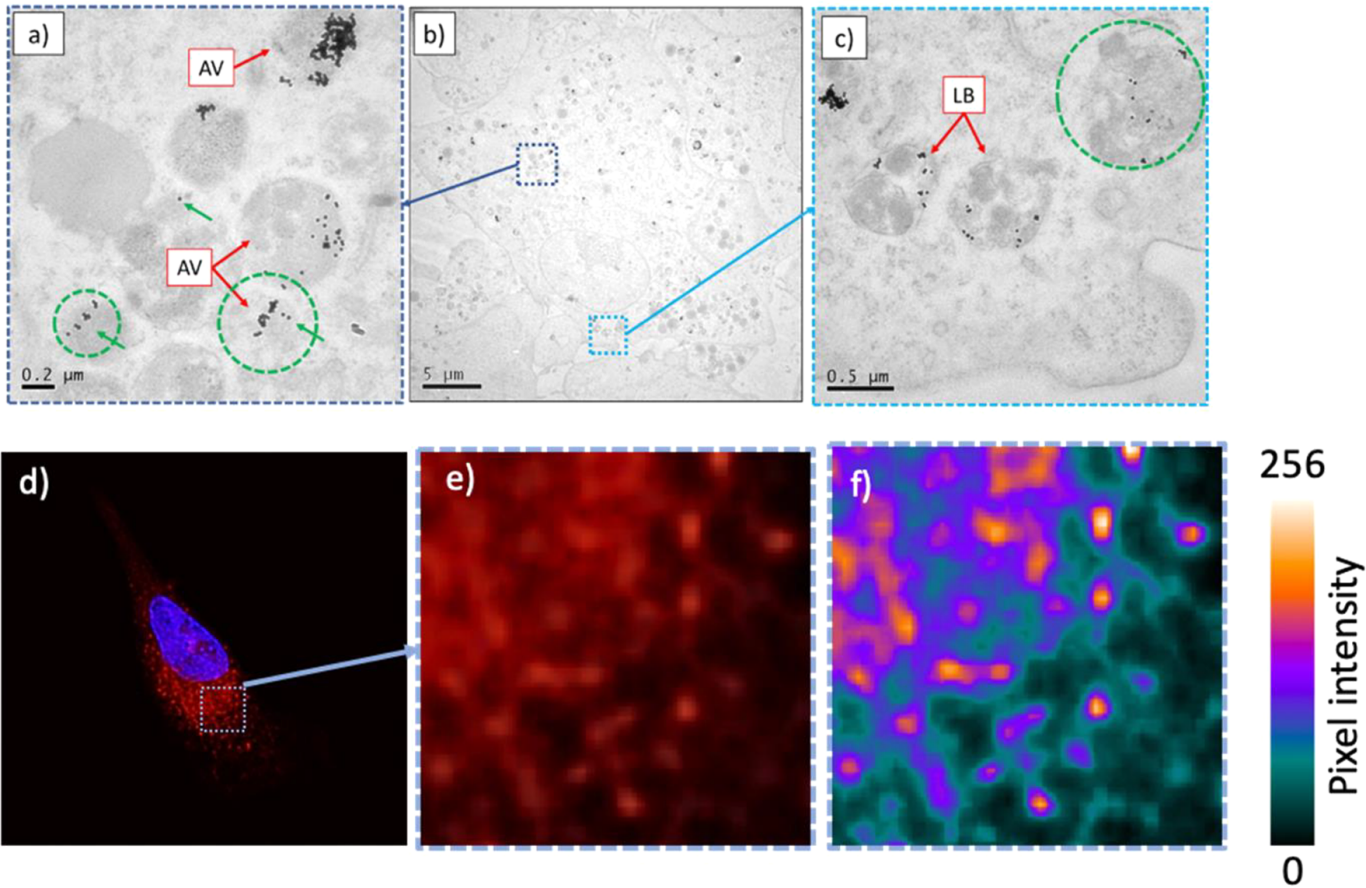
TEM and confocal fluorescence images showing endosomal release of RuS12·AuNPs from lysosomes, multiple vesicular compartments, and autophagosomes into the surrounding cytoplasm of the cell after incubation with 0.9 nM RuS12·AuNPs for 72 h. Individual RuS12·AuNPs are observed in endosomal compartments with very poorly defined, fragmented, and nonexistent membranes (green dotted circles) in (a) and (c), which are zoomed regions of (b). Endosomal escape of the RuS12·AuNPs would release RuS12·AuNPs back into the cytoplasm as indicated by the green arrows in (a). There was also clear evidence of endosomal release of RuS12·AuNPs in cells as observed in confocal microscopy images of the red-luminescence signal of the ruthenium transition metal complex at the same time point, (d) and zoom (e). The red emissive signal of RuS12·AuNPs appears as both punctuate staining associated with particles in vesicles and areas of more diffuse staining in surrounding regions of the cytoplasm, mapped as pixel intensity in (f), which are clearly visible and consistent with endosomal release of RuS12·AuNPs observed by TEM.

We present for the first time, evidence of spontaneous endosomal release of AuNPs from autophagosomes into the cytoplasm in the absence of detectable cytotoxicity. Electron microscopy studies show clear evidence of swelling and disruption of autophagosomes accompanied by endosomal release of RuS12·AuNPs into the surrounding cytoplasm. Although this was first apparent after 48 h of incubation (Figure 4), it was more prominent following 72 h of incubation of cells with RuS12·AuNPs (Figure 7). Furthermore, endosomal release was also detected by confocal microscopy at 72 h and was apparent as areas of diffuse luminescent staining around punctuate foci of RuS12·AuNPs (Figure 7). Image analysis showed clear regions of foci of NP fluorescence (vesicles) and more diffuse regions of staining surrounding vesicles. Although not observed in all cells examined, it is highly suggestive that endosomal release of RuS12·AuNPs can occur. For comparison, identical image analysis of a cell with no evidence of endosomal release is shown in Figure S15. Our data are consistent with previous studies that have used fluorescent probes to assess endosomal release where punctate staining has been reported as an indication of endosomal entrapment and a more diffuse staining pattern as observed here interpreted as evidence of release of material from endosomal vesicles.^13^ Although previously, endosomal release of peptide-coated quantum dots has been observed by confocal microscopy,^45^ to our knowledge, this is the first time that it has been reported for luminescent AuNPs. Previously, Gilleron et al.,^46^ have used 6 nm AuNPs to track endosomal release of 60 nm lipid nanoparticles, and while, this process was very inefficient with only a small fraction of particles able to escape from endosomes, it suggested that with further particle surface modification, it may be possible to enhance this effect.

Although the mechanism of particle release is currently unknown, we propose that it is related to dissolution of gold and chloride ions from the surface of trapped nanoparticles. This would result in an increase in osmolarity within the vesicle causing osmotic stress, influx of water from the surrounding cytoplasm, and subsequent swelling and lysis of autophagosomal membranes. Our observations that RuS12·AuNPs induce changes in the pH of vesicles in the endosomal compartment are consistent with this hypothesis. This mechanism is analogous to the “proton-sponge” mechanism of lysosomal release previously observed with polymeric nanoparticles.^12,13^ In the future, further modifications to the surface of RuS12·AuNPs could be one strategy to enhance further endosomal release of AuNPs in cells, enabling better targeting of nanoparticle cargo to multiple compartments within the cell.

## Conclusion

We have shown that analysis of the luminescence signal from particles in confocal images can be used as a metric of cellular uptake and that it is statistically correlated with total cellular gold content quantified by an analytical ICP-MS method. These results demonstrate a paradigm for using metal-coated, photostable luminescent nanoparticles to quantify cellular uptake. Time-dependent quantification was enabled by the accessibility of the confocal microscopy technique and cellular tracking of the red-luminescent RuS12·AuNPs in a time-resolved manner by tracking the ruthenium luminescence signal. At early time points, there was evidence of free particles in the cytoplasm and mitochondria of cells, which we attribute to diffusion of particles across the cytoplasmic membrane. However, there was direct evidence that the major mechanism of uptake was by macropinocytosis of clusters of particles that had accumulated on the cytoplasmic membrane. Following uptake, particles could be tracked by visualizing their red emissive signal. The majority of RuS12·AuNPs was found located within endosomal compartments of the cell, initially in Rab4-positive early endosomes and at later time points, lysosomes and autophagosomes. Accumulation of particles in autophagosomes was linked with transcriptional and biochemical evidence of active autophagy.

**Figure 8:**
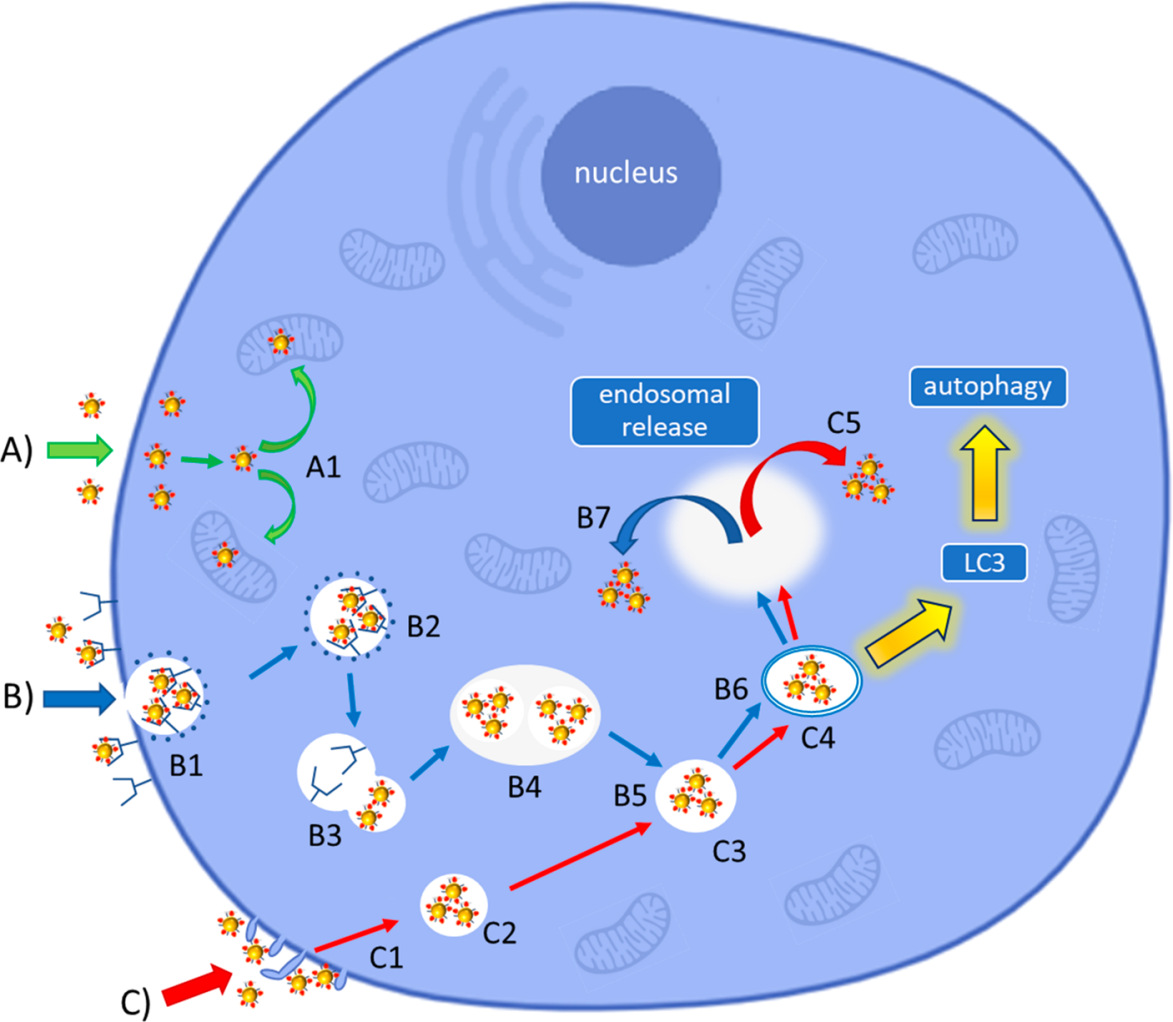
Cellular uptake and endosomal release of RuS12·AuNPs. (A) Direct passive uptake and translocation: (A1) AuNPs pass through the cell membrane into the cytoplasm. Depending on their lipophilicity, the nanoparticles can accumulate in mitochondria. (B) Clathrin-mediated endocytosis: (B1) RuS12·AuNPs bind to protein receptors on the cell membrane and are moved to clathrin-coated pits where the cell membrane folds inward; (B2) clathrin-coated vesicles form; (B3) clathrin-coated vesicles fuse with endosomes; (B4) endosomes traffic the RuS12·AuNP cargo to multivesicular bodies (MVBs); (B5) MVBs fuse with lysomes; (B6) autophagosomal vesicles form and target lysosomal content for degradation, accompanied by the upregulation of LC3; (B7) endosomal escape as the endosomal/autophagosomal vesicle membrane breaks down and nanoparticles are released in to the cytoplasm. (C) Macropinocytosis: (C1) conformational changes in the cell membrane (protrusions and “ruffles”) associated with RuS12·AuNPs; (C2) macropinosomes form to traffic the AuNP cargo; (C3) macropinsomes fuse with lysomes; (C4) autophagosomal vesicles form and target lysosomal content for degradation, accompanied by the upregulation of LC3; (C5) endosomal escape as the endosomal/autophagosomal vesicle membrane breaks down and RuS12·AuNPs are released in to the cytoplasm. Image created in Biorender, https://biorender.com.

Modulation of autophagy by gold nanomaterials has potential therapeutic applications. However, the long-term cellular fate of AuNPs inside autophagosomes remains unresolved and needs to be understood if this is to be developed further. In this study, we show that there was no evidence of any decomposition of RuS12·AuNPs, suggesting that autophagosomes have limited ability to break down AuNPs. Rather, we observed that accumulation of RuS12·AuNPs in autophagosomes was linked to organelle swelling, breakdown of the surrounding membranes, and endosomal release of particles into the cytoplasm (Figure 8). Although further study is required to increase the efficiency of this process, the phenomenon of endosomal release has important consequences for the toxicity, cellular targeting, and therapeutic applications of gold nanoparticles in the future.

## Materials and Methods

Unless otherwise stated, all chemicals and consumables were purchased from Sigma-Aldrich and were of the highest quality available. Additional information about the reagents used is presented in the Supporting Information file

### Citrate-Coated AuNP Synthesis

Synthesis of gold colloids was performed according to the method of Schulz et al.^47^ Briefly, a solution of trisodium citrate (60.0 mg, 0.2 mmol), citric acid (13.3 mg, 0.7 mmol), and ethylenediaminetetraacetic acid (1.0 mg, 0.004 mmol) in 100 mL of deionized water was brought to reflux with vigorous stirring. After 15 min of reflux, there was rapid addition of a preheated solution (80 °C) of HAuCl_4_·3H_2_O (Alfa-Aesar, 8.0 mg, 0.022 mmol) in deionized water (25 mL). After a further 10 min reflux, the heat was turned off, and stirring was continued for an additional 30 min to enable slow cooling to room temperature. The resulting solution of AuNPs (1.6 nM) shows a characteristic surface plasmon resonance (SPR). λ_max_ (H_2_O) = 516 nm, diameter = 14 ± 3 nm (DLS number distribution), PDI = 0.06, ζ potential = −46 ± 16 mV. The concentration of the AuNP colloid was adjusted from 1.6 to 9 nM by centrifugation at 13 000*g* for 30 min and redispersing the pellet in 350 μL of deionized water.

### Ruthenium-Coated Gold Nanoparticles RuS12NP

Coating of the AuNPs was achieved as described previously by Osborne et al.^6^ Briefly, AuNP colloids (1 mL, 9 nM) with the Zonyl FSA fluorosurfactant (10 μL 10% v/v) were monitored by the SPR shift. The solution was centrifuged at 13 000*g* for 30 min, and the pellet was resuspended in 1 mL of water to give Zonyl-coated AuNPs (AuNPs·Z). λ_max_ (H_2_O) = 518 nm (SPR), diameter = 20 ± 5 nm (DLS number distribution), PDI = 0.05, ζ potential = −50 ± 8 mV. A solution of RuS12 (20 μL, 0.87 mM) was titrated as 2 μL aliquots into 1 mL of AuNPs·Z to give RuS12·AuNPs. Size-exclusion chromatography (Sephadex G25) was used to isolate the pure RuS12·AuNPs. λ_max_ (H_2_O) = 520 nm (SPR), diameter = 18 ± 5 nm (DLS number distribution), PDI = 0.22, ζ potential = −42 ± 15 mV.

### Cell Culture

A549 human lung adenocarcinoma epithelial cells (86012804) were purchased from the European Collection of Authenticated Cell Cultures. Cultures were grown as a monolayer in a humidified atmosphere (5% CO_2_ incubator; 95% air) at 37 °C. Cells were maintained by growing in a vented cap T_75_ flask at 37 °C in Dulbecco’s modified Eagle medium (DMEM) supplemented with 10% fetal bovine serum (FBS), 2 mM l-glutamine, 100 U/mL of penicillin, and 100 μg/mL of streptomycin. Media was replaced every 2–3 days, and cells were subcultured at approximately 70–80% confluence using a standardized trypsin–EDTA protocol. All cell cultures were confirmed free from *Mycoplasma sp*. contamination using the EZ-PCR mycoplasma detection kit according to the manufacturer’s instructions. All cells were cultured up to passage 20 before being discarded.

### Treatment of Cells with AuNPs

Uptake of RuS12·AuNPs into A549 cells was studied over a period of 2–72 h. The final concentration of particles used (0.9 nM) has been previously shown to be noncytotoxic to cells, and this was confirmed in the current study.^9^

### Confocal Microscopy Imaging of Cells

Cells were seeded at a density of 100 000 into a 35 mm dish with a 10 mm glass diameter insert (Matek) and allowed to attach overnight. Following treatment with RuS12·AuNPs, cells were incubated with Hoechst 33258 (2.5 μg/mL, 30 min). For live cell imaging, cells were finally rinsed three times with PBS and imaged in live cell imaging solution (Thermo Fisher). For fixed cells, cells were rinsed three times with PBS followed by fixation with paraformaldheyde (4%, 15 min) at room temperature. Next, cells were further rinsed two times with PBS and stained with 1 μg/mL of Hoechst 33258 for 10 min. Cells were then rinsed twice with PBS followed by mounting to a glass slide with a drop of Hydromount media (National Diagnostics). Uptake of RuS12·AuNPs into A549 cells by confocal microscopy was investigated using a Leica SP2 confocal system with 63× and 100× oil immersion objective lenses. Images were acquired in fluorescence, reflectance, and transmission mode. Fluorescence channels were acquired with the following excitation and emission values: Hoechst (blue channel): λ_exc_ = 405 nm (75%), λ_em_ = 410–455 nm, nanoparticle luminescence (red channel): λ_exc_ = 458 nm (100%), 476 nm (100%), 488 nm (100%), 496 nm (100%), and 514 nm (57%), λ_em_ = 620–800 nm, and GFP (green channel) λ_exc_ = 488 nm, λ_em_ = 502 nm. Reflection images were acquired at λ_exc_ = 488 nm (67%) and λ_em_ = 478–498 nm, and transmission images were acquired using the default transmission setup of the microscope with a beam intensity of 1–3%. All images acquired were processed by imaging software (ImageJ Version 1.43M).

### Transmission Electron Microscopy

Cells were seeded at a density of 100 000 cells per well on to a sterilized 13 mm diameter coverslip in a six-well plate and allowed to attach overnight. The next day, cells were rinsed with PBS and then treated with 0.9 nM RuS12·AuNPs for 2, 4, 8, 24, 48, and 72 h. At the end of each time point, media was aspirated, and cells were rinsed three times with PBS followed by fixation with 2.5% glutaraldehyde for 24 h at 4 °C. Samples were taken for processing at the Centre for Electron Microscopy (University of Birmingham). Ultrathin sections of between 70–90 nm were cut parallel to cover glass and mounted onto Formvar-coated 200 mesh copper grids. Images were acquired with a JEOL 1200 EX transition electron micrograph operated at 80 kV in imaging mode. Images were acquired using Digital Micrograph Version 1.83.842.

### Inductively Coupled Plasma Mass Spectrometry

Cells were seeded at a density of either 100 000 cells per well in a six-well plate or 3 × 10^6^ cells in a T_75_ flask (for Ru determination) and left overnight for attachment to occur. Media was aspirated and replaced with 3 mL of complete media (10 mL for T_75_ flasks) containing 0.9 nM RuS12·AuNPs and treated for 2, 4, 8, 12, 16, 24, 32, 40, 48, 56, 64, and 72 h. At the end of each time point, media was removed, and cells washed three times with 1 mL of PBS (10 mL for T_75_ flasks). Cells were detached by treating with 1 mL of trypsin (10 min) and pelleted by centrifuging for 10 min at 1500*g*. Cell pellets were digested in 300 μL of ultrapure aqua regia (3HCl/1HNO_3_) overnight at room temperature. The next day, digested samples were diluted with 4% HNO_3_ to reduce the aqua regia content to less than 4%, and samples were analyzed at an analytical chemistry lab at the University of Warwick. The ICP-MS experiment was done as a biological triplicate with three technical triplicates. A series of standard solutions of gold (0, 0.2, 0.5, 1, 2, 5, 10, 20 ppb) were used for calibration and determining metal content.

### Labeling of Cells with Organelle Specific Probes

GFP-tagged organelle specific markers (Origene) were visualized in cells by transiently transfecting plasmid DNA into A549 cells. In addition, Golgi, endoplasmic reticulum, and mitochondria were labeled with Golgi-ID, ER-Tracker Green, and MitoGreen, respectively. To assess lysosomal pH, cells were labeled with LysoSensor Blue. Additional experimental details about these reagents are presented in the Supporting Information file.

### LC3 Western Blotting and qPCR

LC3 was analyzed following treatment of cells with RuS12·AuNPs at both the protein level by Western blotting and at the mRNA level by qPCR. Additional experimental details are provided in the Supporting Information file.

## Supporting Information Available

Characterization of RuS12NP, lack of cytotoxicity, additional supporting mass spectrometry data, images of RuS12NP in fixed cells, details of the particle quantification method, and additional supporting biochemical evidence of mechanism of uptake and lysosomal releases (PDF)
