# Supplementary material for: Quantification by Luminescence Tracking of Red Emissive Gold Nanoparticles in Cells
Source: JACS Au. 2021 Jan 19;1(2):174–86. doi: 10.1021/jacsau.0c00033 (PMC7990080; doi:10.1021/jacsau.0c00033)
Supplement: Supplementary file 1 — au0c00033_si_001.pdf [file au0c00033_si_001.pdf]

Supporting Information

Quantification by luminescence tracking of red emissive gold nanoparticles in cells

Abiola N Dosumu[1†], Sunil Claire[2†], Luke S Watson[2], Patricia M Girio[2,3], Shani A M Osborne[2], Zoe Pikramenou[2] and Nikolas J Hodges[1]*

[1]School of Biosciences, [2]School of Chemistry and [3]Doctoral Training Centre in Physical Sciences for Health, The University of Birmingham, Edgbaston, Birmingham, B15 2TT, United Kingdom.

*Corresponding author: N.Hodges@bham.ac.uk

[†] Both authors contributed equally to this work

This PDF file includes:

Supporting Figures S1 to S15

Additional Materials and Methods Section including information on reagents used

A)

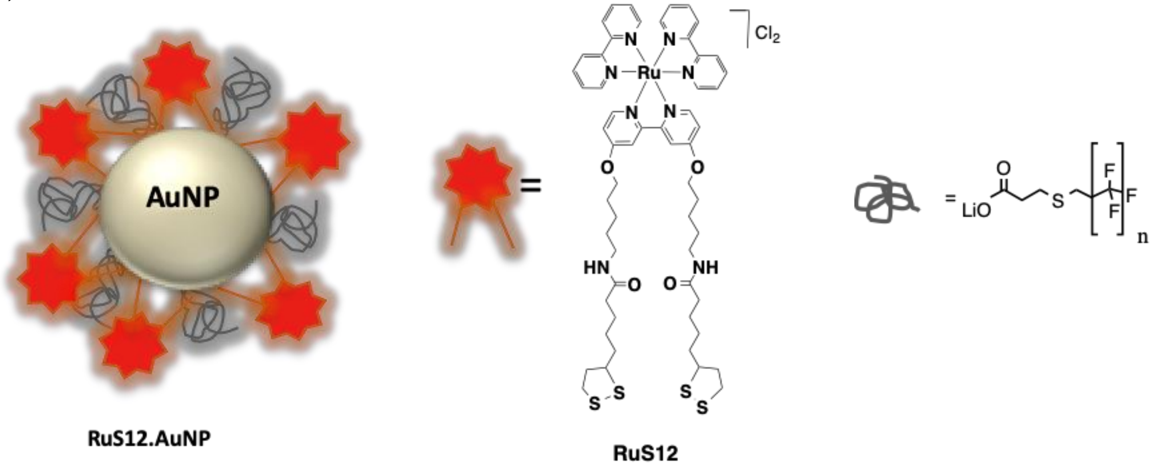

B)

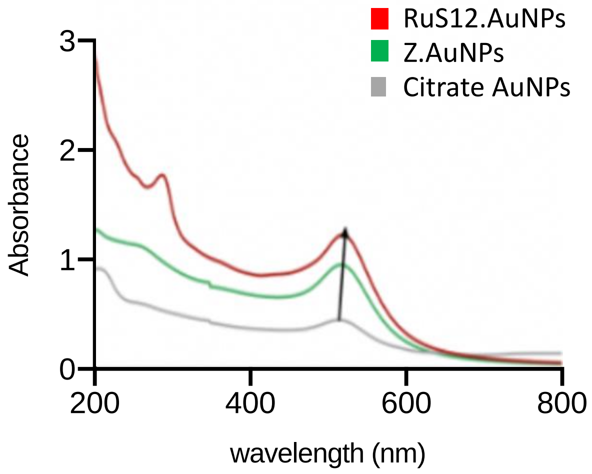

C)

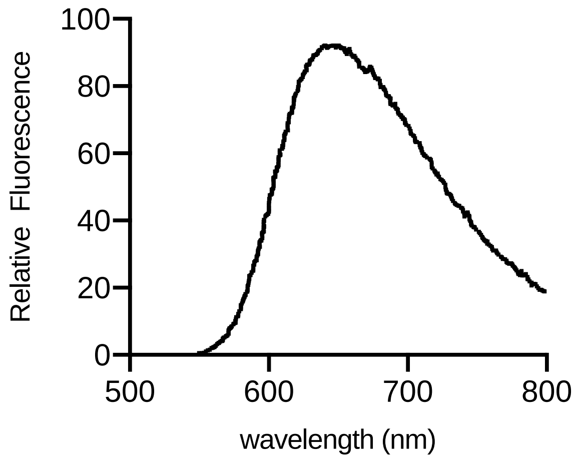

D)

Sample	λmax (nm)	Shift (nm)	Number distribution	Intensity (nm)	Distribution (PDI)
Citrate stabilized AuNP colloid	516	0	14± 3	20±5	0.06
Z.AuNP	518	2	20±5	27±7	0.05
Purified RuS12.AuNP	520	4	18±5	36±15	0.22

E)

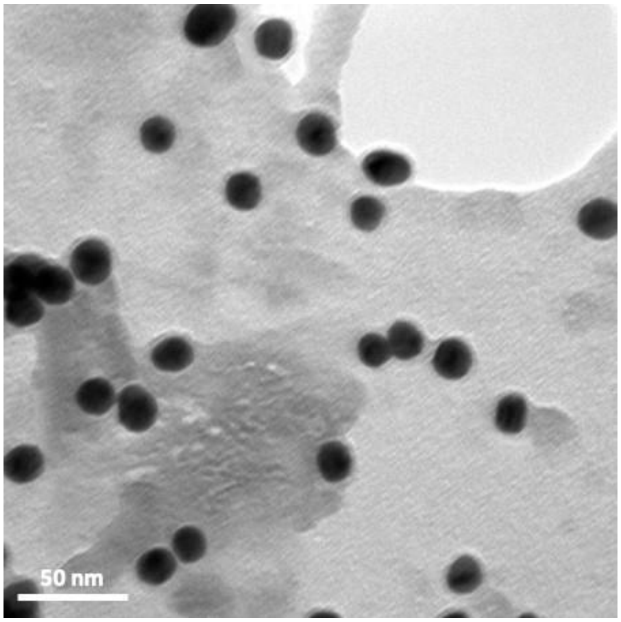

F)

<u>Number of gold atoms, *N* in a gold nanoparticle</u>

Assuming nanoparticles are comprised of perfect spheres, atoms are packed in a uniform face-centred cubic crystalline structure, and taking the density of gold as ρ_{gold} = 19.32 g cm^{-3}

$$N = \frac{\rho \ \times \pi D^3}{6 \times Mr} \times N_A$$

where Mr =196.96657 g mol^{-1} , N_A = 6.022140857 × 10^{23} mol^{-1}

$$\therefore N = \ 30.8969 \times D^3$$

Figure S1: Nanoparticle schematic and characterization. **A)** Schematic of RuS12.AuNP, **B)** UV-Vis absorption spectra showing the shift in the SPR band due to functionalization of the surface of AuNP. **C)** Luminescence emission spectra of RuS12.AuNP showing the characteristic ruthenium red signal with a peak at 650 nm (λexc = 488 nm). **D)** Summary of SPR shifts and DLS data. **E)** Transmission electron microscopy of RuS12.AuNP, the diameter and standard deviation of the gold core calculated from 14 randomly selected particles was 15.5±1.0 nm (95% confidence interval values 14.9–16.11 nm, coefficient of variation 6.6%). **F)** Calculation of the number of gold atoms in a nanoparticle.

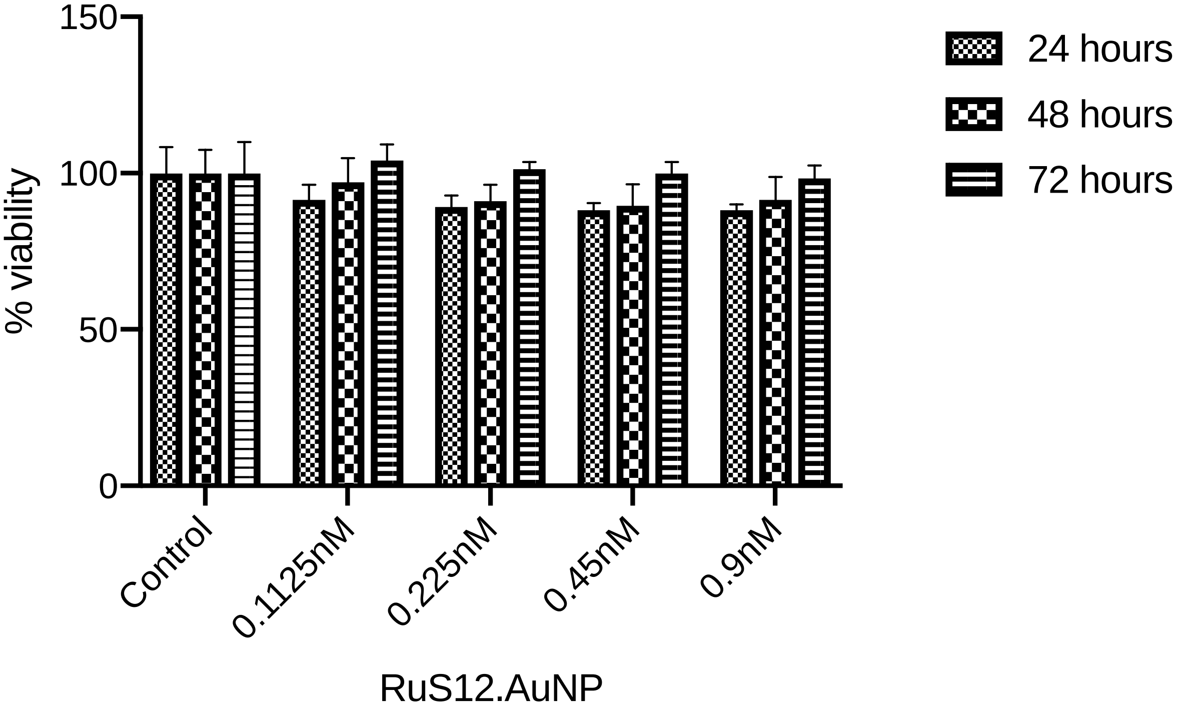

Figure S2: Lack of cytotoxicity of RuS12.AuNPs as assessed by the MTT reduction assay. Cells were seeded at 8000 cells per plate in 96-well plates and left overnight for attachment to occur. The next day the media was removed and fresh media (100 μL) containing RuS12.AuNPs added. Following treatment with RuS12.AuNPs for 24, 48 and 72 hours, the media was removed, and cells incubated with 100 μL of media containing 0.5 mg mL^{-1} MTT solution for 3 hours. The media was removed and reduced MTT was solubilized by addition of DMSO (100 μL) and absorbance at 590 nm measured in a plate reader (Tecan Infinite 200 Pro) as an indicator of mitochondrial reductive activity and cellular viability. All samples were normalized to a solvent (DMSO) blank and the results represent the mean of three experiments carried out in triplicate (n=3±SEM).

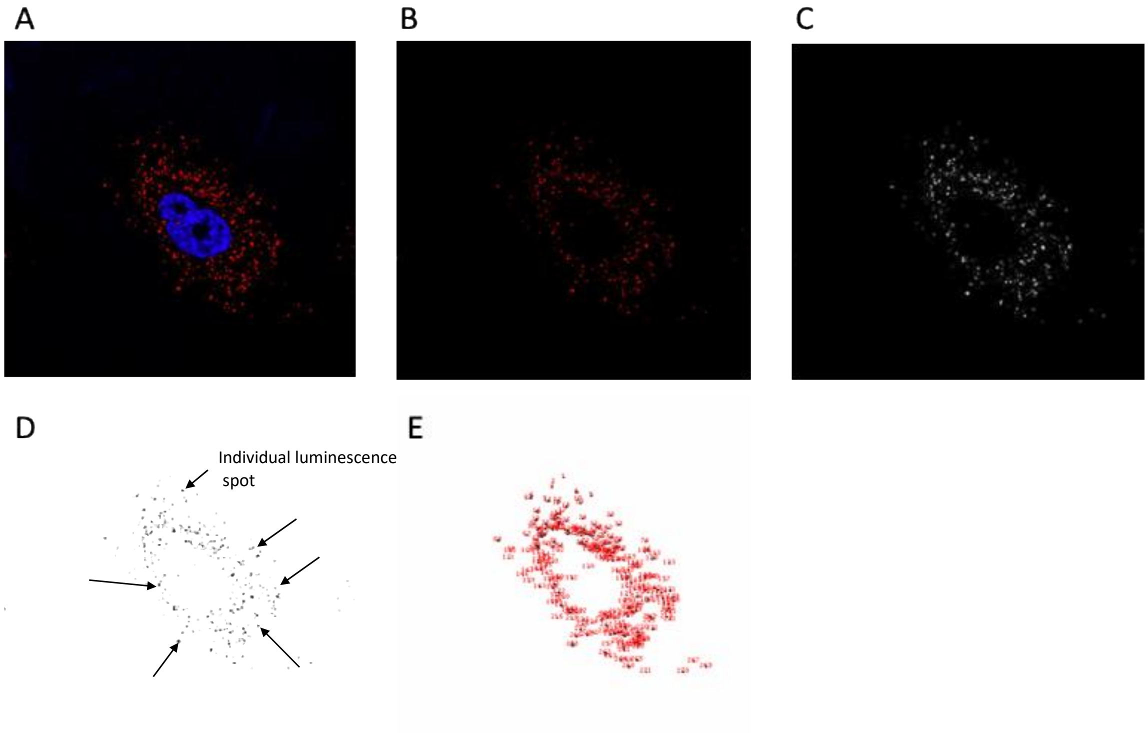

Figure S3: Outline of workflow for the quantification of number of luminescence spots in cells: A) Confocal images are acquired and the red channel extracted (B) and converted to greyscale (C). The greyscale image then undergoes thresholding using the threshold function in image J to produce a binary image to identify individual luminescence spots (examples indicated by arrows). (D). Finally, the binary image is then analysed using the analyse particle tools on Image J to count the total number of individual features in the image (E).

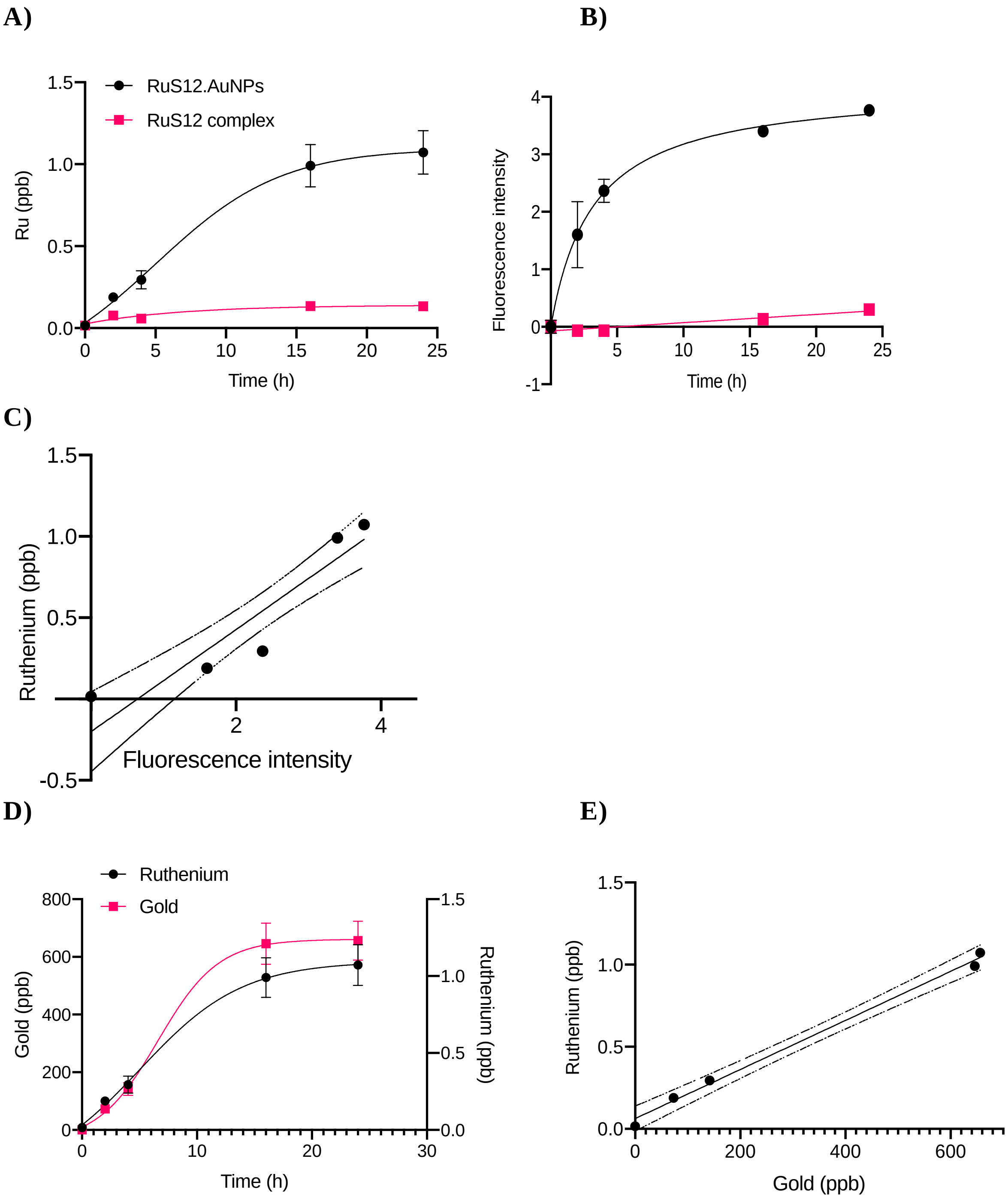

Figure S4: Time-dependent accumulation of cellular ruthenium-content as quantified by **A)** ICP-MS and **B)** fluorescence using flow cytometry (λexc = 488 nm, λem >670 nm, long pass filter) in cells treated with either 0.9 nM RuS12.AuNPs or 0.6 µM free RuS12 complex for 2–24 hours. **C)** Linear regression analysis between cellular ruthenium-content as assessed by ICP-MS (y-axis) and Ruthenium complex fluorescence intensity as assessed by flow cytometry (x-axis) in cells treated with RuS12.AuNPs. There was a statistically significant linear correlation ($P<0.001$, $F=56.6$, $R^2=0.85$). The dotted lines indicate 95% confidence boundaries for the line of best fit. **D)** Comparison of cellular uptake of RuS12.AuNPs as assessed by ICP-MS quantification of Gold and Ruthenium. **E)** Linear regression analysis between cellular Ruthenium (y-axis)- and Gold (x-axis)-content as assessed by ICP-MS There was a statistically significant linear correlation ($P<0.0001$, $F=344.0$, $R^2=0.97$). The dotted lines indicate 95% confidence boundaries for the line of best fit.

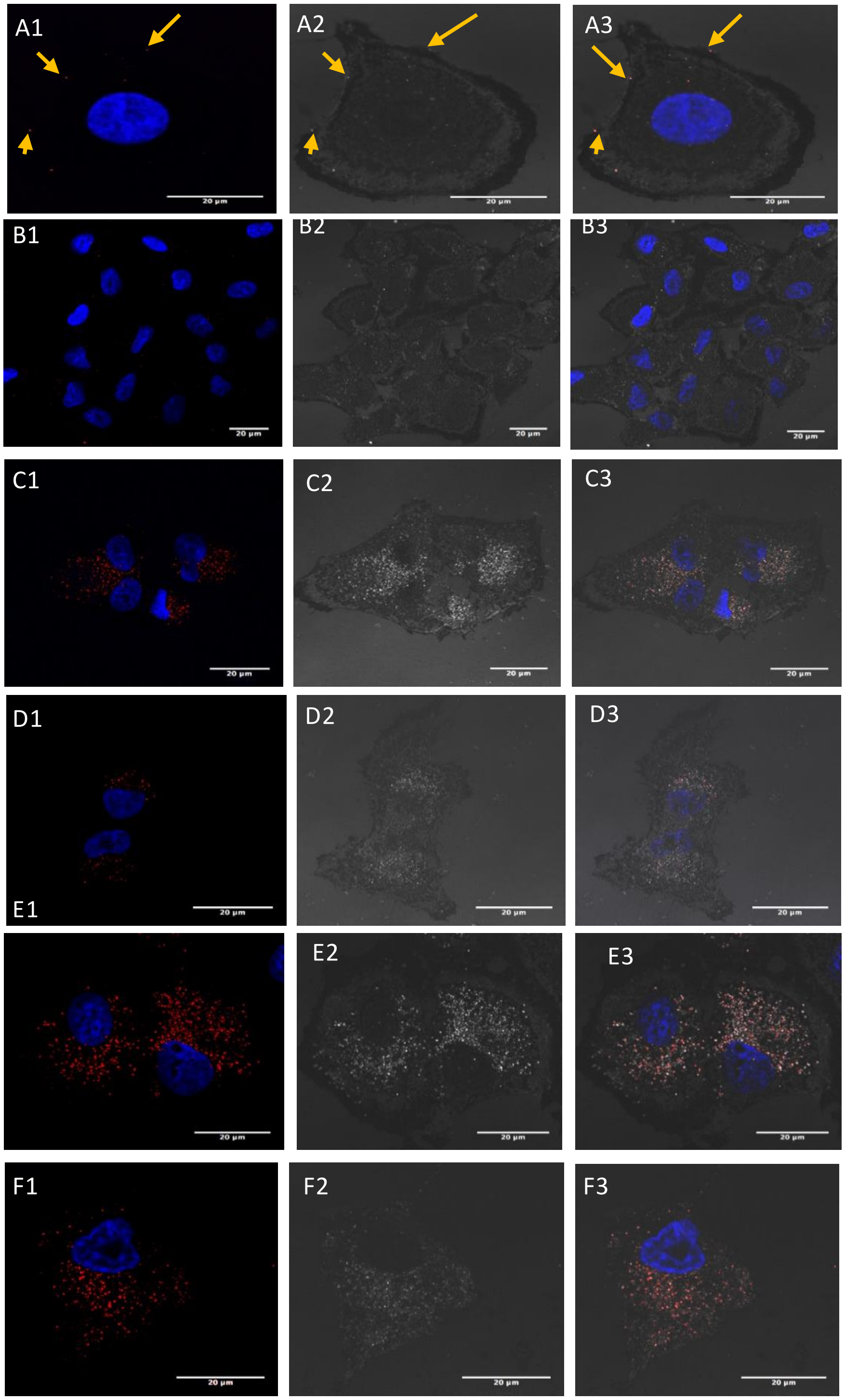

Figure S5: Confocal images of fixed A549 cells treated with 0.9 nM RuS12.AuNP and counterstained with Hoechst. A= 2 hours, B= 4 hours, C=16 hours, D=24 hours, E=48 hours and F=72 hours. 1. Merged images of RuS12.AuNP and Hoechst luminescence channels 2. Corresponding reflection image (λexc = 488 nm, λem = 478-498 nm)3. Merged fluorescence and reflection channel. Ruthenium emission from red channel (λexc = 488 nm, λem = 620-800 nm) Hoechst emission from the blue channel (λexc = 405nm, λem = 410-455 nm). Scale bar on all images is 20 µm.

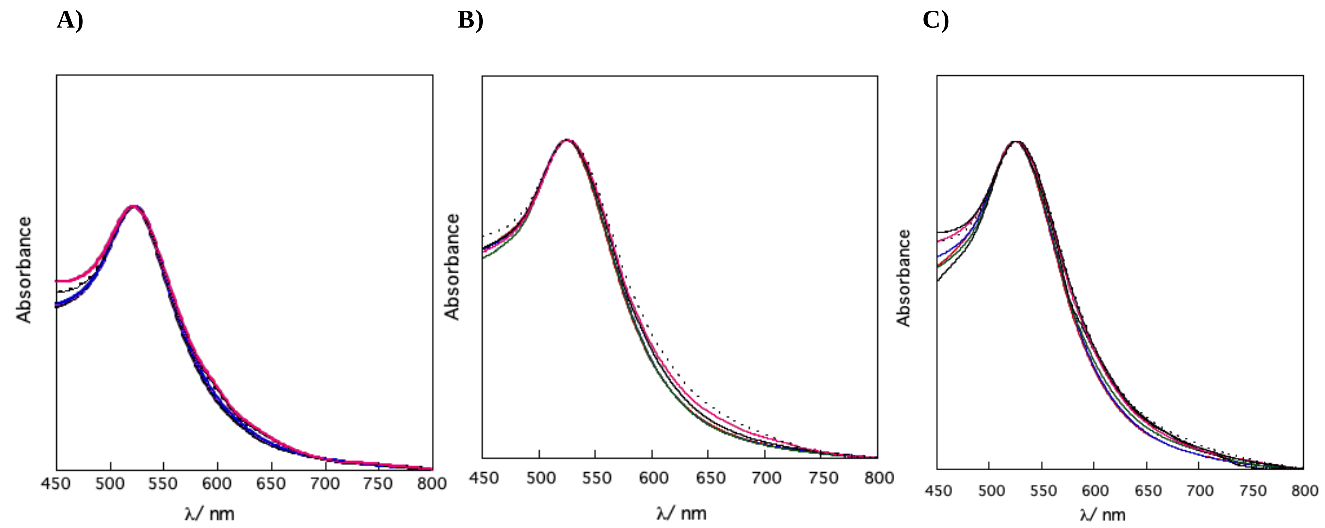

Figure S6. Normalised UV-vis spectra showing SPR band of 0.9 nM of **RuS12.AuNP13** at different time points to show stability of particles: A) in cell media at 0, 2,4,16, and 72 hr , B) in cell media with 5mM glutathione at 0, 2, 4, 20, 24, 48 hr and C) in cell media with 10 mM glutathione at 0, 2, 4, 20, 24, 48, 72 hr. The supernatant of the particles showed no detectable ruthenium emission confirming the stability of the particles.

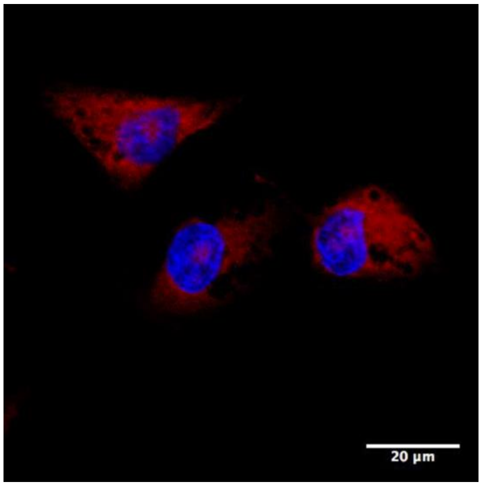

Figure S7: Image of free RuS12 molecular probe (0.63 μM) in cells treated for 4 hours. Red channel, Ruthenium emission (λexc = 488 nm, λem = 620-800 nm). Blue channel, Hoechst emission (λexc = 405nm, λem = 410-455 nm). Scale bar is 20 μm.

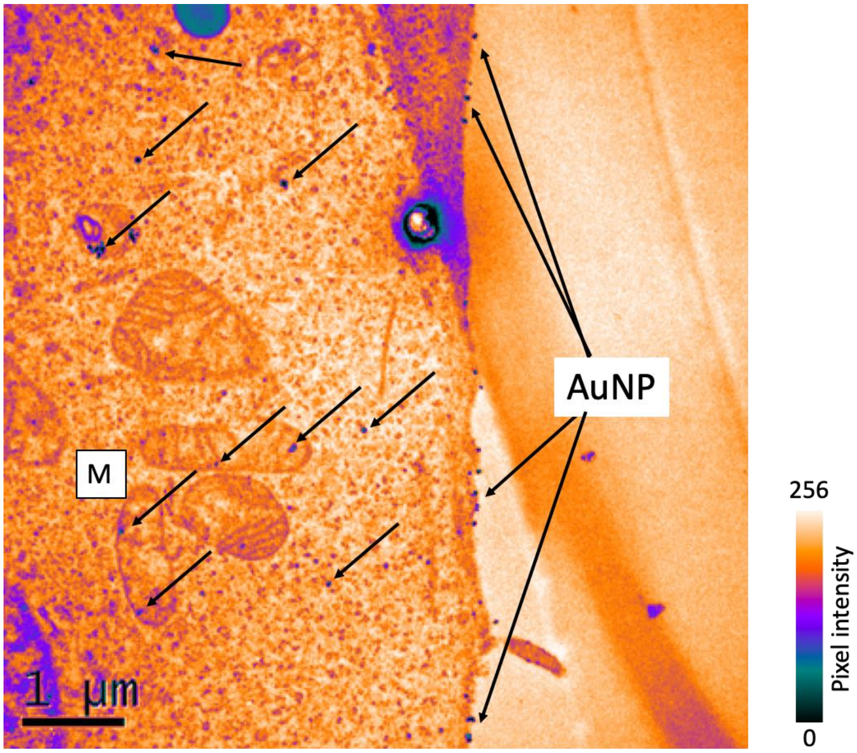

Figure S8: False color image of electron micrograph reproduced from Figure 3A showing optical dense spherical features in both the mitochondria and cytoplasm that are the same as those on the membrane and thus particles distinct from less dense background granular staining of the cytoplasm and therefore interpreted as RuS12.AuNP.

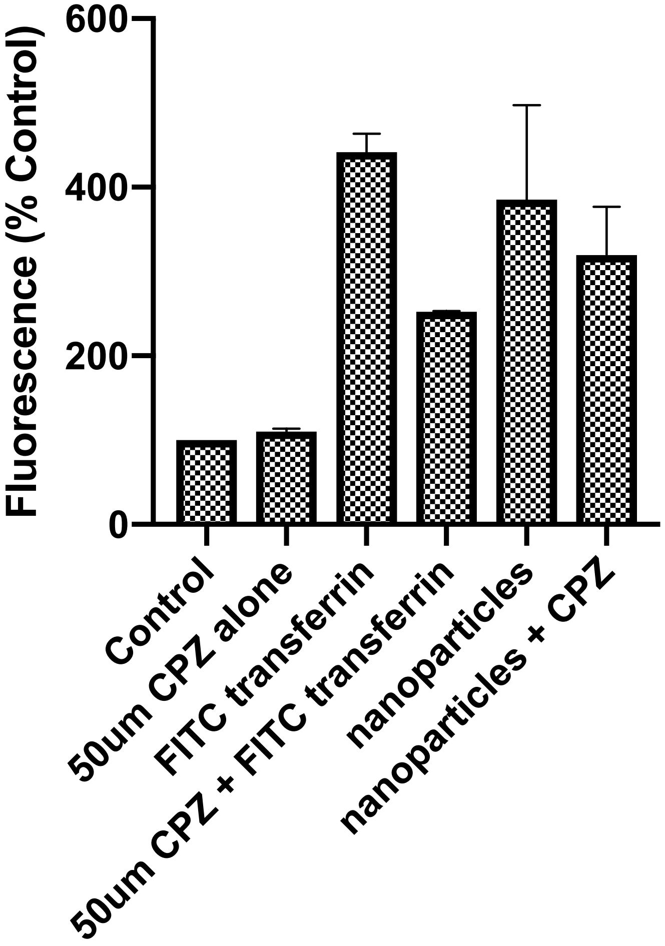

Figure S9: Chlorpromazine (CPZ) pre-treatment (50 μM, 30 minute) inhibits cellular uptake of FITC-labelled transferrin but not RuS12.AuNP. The mean fluorescence of 10 000 cells was quantified by flow cytometry and normalised to the untreated control. The results represent the mean of three independent experiments ±SD (n=3).

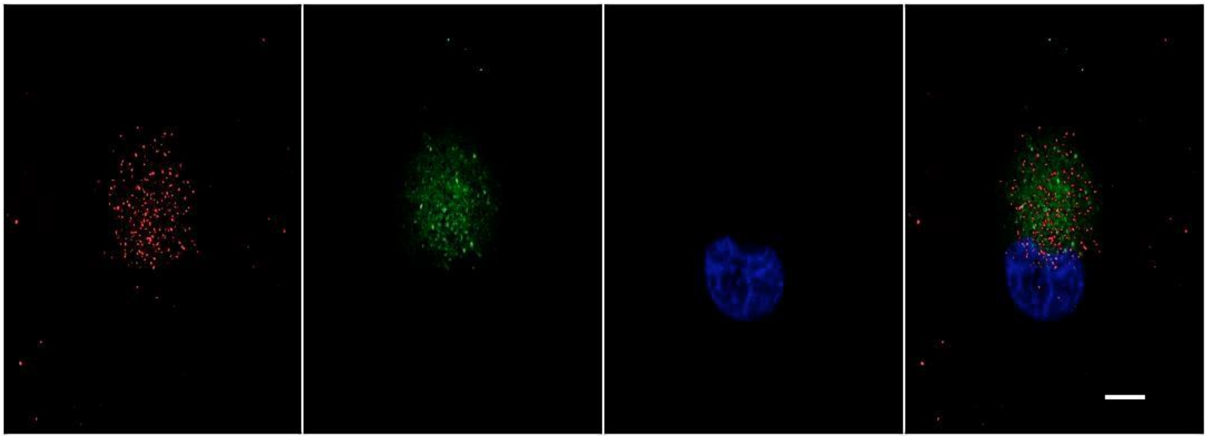

Figure S10: Confocal luminescence imaging to investigate overlap with mitochondria after at 24h incubation with 0.9 nM RuS12.AuNP. Red channel, RuS12.AuNP emission (λexc = 488 nm, λem 620-800 nm), green channel, GFP emission represented signal from mitotracker green (λexc = 488 nm, λem = 502 nm), blue channel, Hoechst emission (λexc= 405 nm, λem = 410-455 nm). Scale bar is 20 μm.

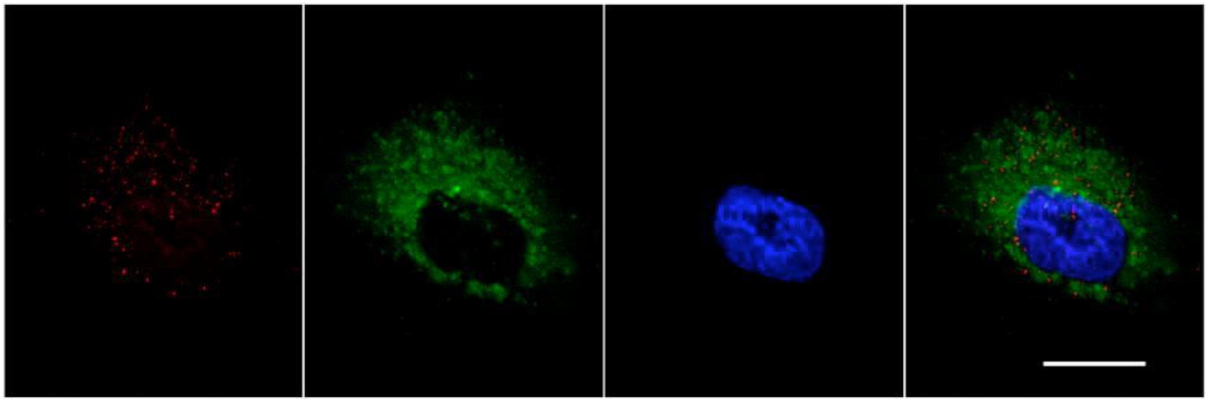

Figure S11: Lack of co-localization with the golgi-apparatus at 24h. Red channel, RuS12.AuNP emission (λexc = 488 nm, λem 620-800 nm), green channel, GFP emission represented signal from Golgi-ID (λexc = 488 nm, λem = 502 nm), blue channel, Hoechst emission (λexc= 405 nm, λem = 410-455 nm). Scale bar is 20 μm.

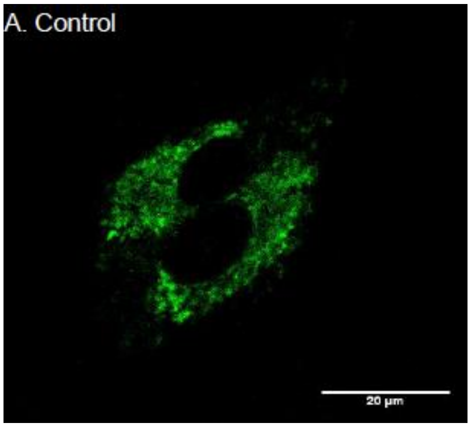

Figure S12: Diffuse LC3-GFP staining in control cells with lack of punctuate foci indicating lack of active autophagosomes. Green channel, GFP emission represented signal from GFP (λexc = 488 nm, λem = 502 nm). Scale bar is 20 μm.

A)

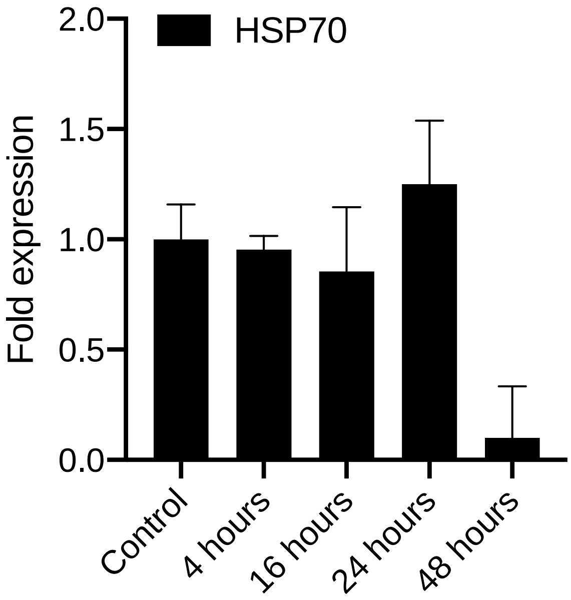

B)

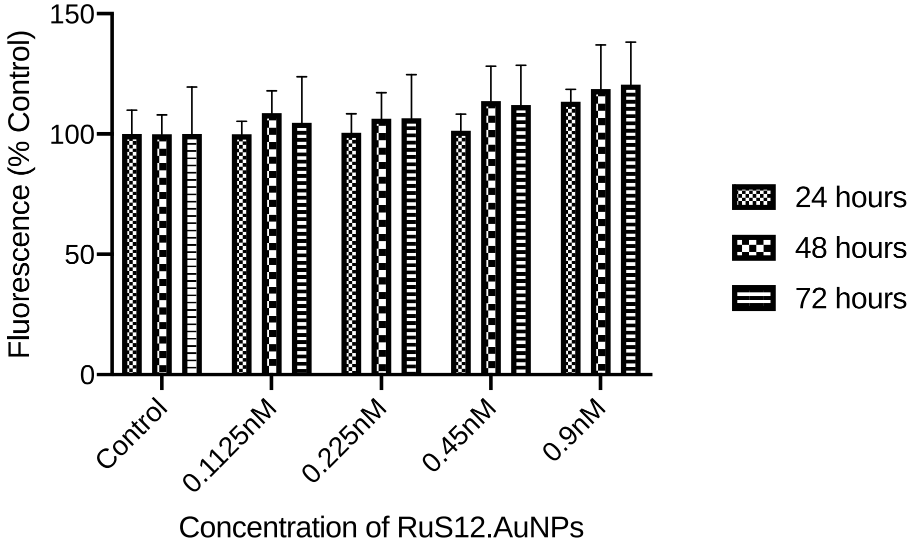

Figure S13: A) Lack of transcriptional activation of HSP70, A549 cells were treated with 0.9 nM RuS12.AuNP for 4, 16, 24 and 48 hours. At the end of each time point, RNA was extracted and reverse transcribed to cDNA. cDNA was used for RT-PCR with TaqMan gene expression. The experiment is a biological triplicate for each time point with three technical replicates ±SD (n=3). B) 0.9 nM RuS12.AuNP (0.9 nM) do not induce oxidative stress as assessed by DCF-DA oxidation at any of the timepoints investigated. Cells were seeded in 96-well in white clear bottomed 96-well plates (8000 per well) and left overnight for attachment to occur. Following treatment, cells with incubated with DCF-DA (10 μM, 45 min) before fluorescence intensity was measured in a plate-reader (Tecan Infinite 200 Pro, λexc 485 (30 nm bandwidth), λem 535 (30 nm bandwidth). The experiment is a biological triplicate for each time point with three technical replicates (n=3±SEM).

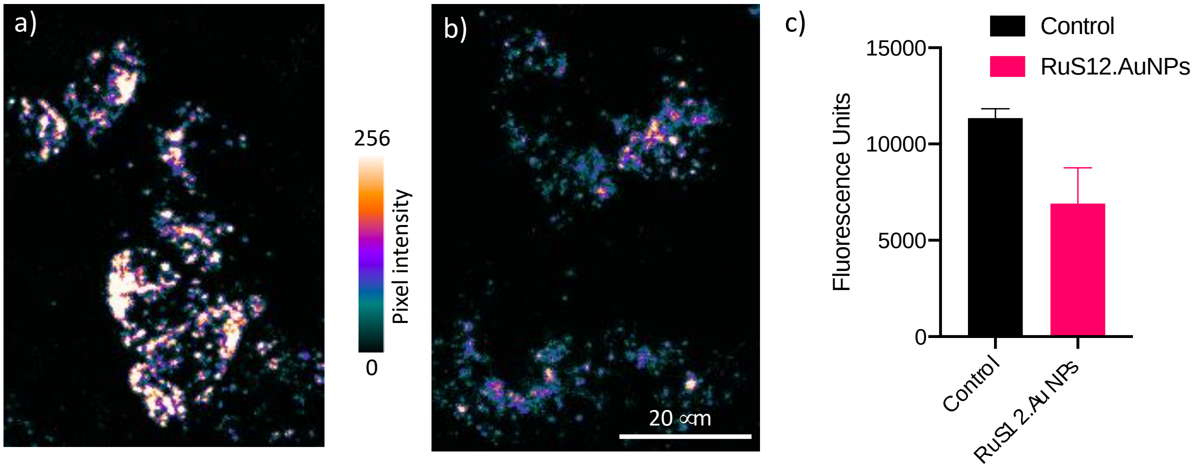

Figure S14: Increase in lysosomal pH as assessed by loss of lysoSensor Blue (1 µM, 1h) fluorescence (λexc 405 nm, λem 420-460 nm) following treatment of cells with 0.9 nM RuS12.AuNPs for 72h. A) Control cells, B) 0.9 nM RuS12.AuNP treated cells, scale bar represents 20 µm. C) Plate reader (Tecan Infinite 200 Pro) quantification of LysoSensor Blue fluorescence, λexc 360 (30 nm bandwidth), λem 465 (30 nm bandwidth) in control and 0.9 nM RuS12.AuNP treated cells.

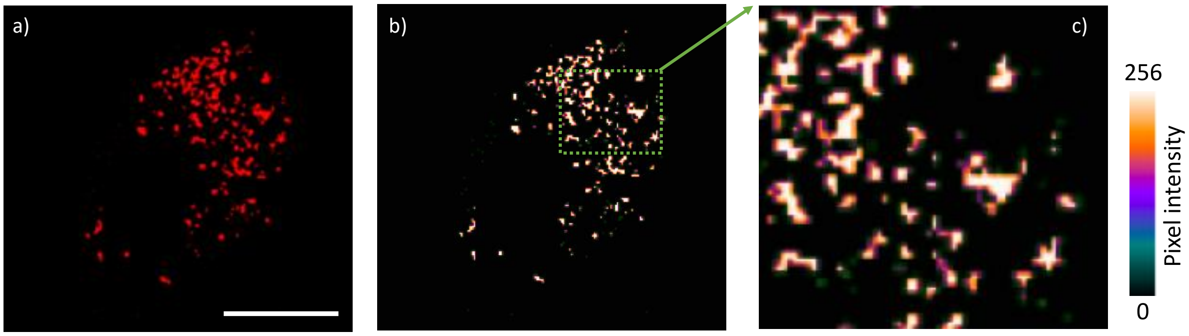

Figure S15: Example of cell taken from Figure 7 with no endosomal release of AuNPs. A) red RuS12.AuNP fluorescence, B) false color pixel intensity image generated in the same way as figure 7F, C) zoom showing that particles in this cell are restricted entirely to the endosomal compartment. Scale bar is 20 μM.

Data Availability Statement
All additional data and information is included in the supporting information file as supporting figures. In addition, additional information about experimental details and reagents used are also provided.

Additional experimental information

Additional information about cell tracking reagents:
The plasmids used for expression of GFP-tagged organelle specific markers in this study (LAMP1, Rab4, LC3) are commercially available from Origene https://www.origene.com/products/cdna-clones/organelle-marker.

Transfection of cells
A549 cells were seeded at a density of 60,000 in a 6-well plate and left to attach overnight. The next day cells were transfected according to manufacturer instructions (Origene). Briefly, 9 µL of Turbofectin 8.0 (Origene) was added to 300 µL of serum free DMEM followed by the addition of 3 µg of isolated plasmid DNA and gently pipetting of mixture. The transfection mixture was incubated at room temperature for 25 minutes followed by drop-wise addition to cells. Plates were gently shaken to ensure homogenous transfection and left to incubate at 37C for 48 hours. 48 hours post-transfection, cells were treated with 0.9 nM RuS12·AuNPs. After treatment, cells were fixed with 4% PFA, and in some cases counterstained with Hoechst 33258 (1 µg/mL) and mounted on a glass slide for confocal imaging.

Labelling of organelles with chemical probes
Additional colocalization studies were undertaken with the following commercially available organelle specific probes:

Golgi-ID (http://www.enzolifesciences.com/ENZ-51028/golgi-id-green-assay-kit/)
ER-Tracker green (https://www.thermofisher.com/order/catalog/product/E34251#/E34251)
MitoGreen (https://www.thermofisher.com/order/catalog/product/M7514#/M7514)

A549 cells were grown on coverslips in 6-well plates (60 000 cells per well), following incubation with RuS12.AuNPs cells were labelled with organelle specific probes at the following final concentrations of Golgi-ID, 1 µM (30 min), ER-tracker green, 1 µM (30 min) and MitoGreen 200 µM (10 min) respectively. In some cases, cells were counterstained with Hoechst 33258 (1 µg/mL) prior to analysis by confocal microscopy. LysoSensor Blue (https://www.thermofisher.com/order/catalog/product/L7533#/L7533) was used to assess lysosomal pH in cells (1 µM, 60 min).

Western blotting protocol:
For LC3 western blotting the primary antibody (#2775) used is used is commercially available from Cell Signaling Technologies, https://www.cellsignal.co.uk/products/primary-antibodies/lc3b-antibody/2775. The secondary antibody used was sheep anti-rabbit HRP conjugated from BioRad, this product has been discontinued.

For beta-actin western blotting the primary antibody used (clone AC-74) is commercially available from Sigma, https://www.sigmaaldrich.com/content/dam/sigma-aldrich/docs/Sigma/Datasheet/6/a2228dat.pdf. The secondary antibody used was goat-anti mouse HRP conjugated commercially available from ThermoFisher (product number A16066). https://www.thermofisher.com/antibody/product/Goat-anti-Mouse-IgG-H-L-Secondary-Antibody-Polyclonal/A16066.

Cells were seeded at a density of 400,000 in a T_{25} flask and allowed to achieve approximately 60% confluence before treatment with 0.9 nM RuS12·AuNP for 2, 4, 8, 12, 16 and 24 hours. At the end of each time point, cells were washed three times with PBS and trypsinised with 0.5 mL of trypsin for 5 minutes at 37°C. The supernatant was discarded, and the pellet stored at -80°C until required. Frozen pellets were thawed and incubated on ice with regular vortexing and pipetting for 20 minutes with 250 µL of radio-immunoprecipation (RIPA) buffer (1 M Tris-HCl pH 7.4, 150 mM NaCl, 1% Triton X-100, 0.5 M EDTA, 10% w/v sodium deoxycholate and 10% SDS) supplemented with 0.01 % mammalian protease inhibitor

cocktail (Sigma). After 20 minutes, cells were centrifuged for 15 minutes at 14,000 rpm and 4°C and the supernatant retained. Total protein (40 µg) was mixed with an equal volume of 2x Laemelli sample buffer and denatured by heating at 95°C for 5 minutes. Samples were then loaded into wells made with 4 % stacking gel (4% acrylamide, 125 mM Tris-HCl pH 6.8, 0. 1% SDS, 1µL/mL TEMED and 10 µL/mL ammonium persulfate) and 12. 5% resolving gel (12.5% acrylamide, 375 mM Tris-HCl pH 8.8, 0.1% SDS, 1.5 µl/mL TEMED and 15 µL/mL ammonium persulfate) and samples ran for 90 minutes at 120V. Separated proteins were transferred onto 0.2 µm PVDF membrane (Bio-Rad Trans-Blot® Turbo™ Mini PVDF) using a Trans-Blot® Turbo™ transfer system (Bio-Rad). The PVDF membrane was blocked with blocking buffer 5% (w/v) BSA in [0.1% TBST (Tris buffer Saline and 0.1% Tween-20)] for 1 hour on rocker at room temperature to stop any non-specific binding. This was followed by overnight incubation with primary antibody LC3 raised in rabbit (cell signalling technology) diluted with blocking buffer 1:1000 and ß-actin raised in mouse (used as housekeeping control genes) diluted with blocking buffer 1:10,000 all at 4°C with gentle shaking. Membranes were washed three times for 10 minutes each, with 0.1% TBST on a rocker at room temperature, before incubating with secondary antibody. Secondary antibodies used were all horseradish peroxidase (HRP) conjugated and diluted with blocking buffer 1:5000 with sheep anti-rabbit antibody (Bio-Rad) for LC3 primary protein and 1:1000 with goat anti-mouse for ß-actin (ThermoFisher) and incubate at room temperature for 1 hour on a rocker. Membranes were washed twice for 10 min with TBST and once with TBS before protein detection using SignalFire™ ECL Reagent (Cell Signalling Technology) and Amersham Hyperfilm (GE healthcare). The film was exposed for 5 minutes and developed using a XoGraph machine (AGFA Curix 60).

qPCR protocol

All primers used were purchased from Thermofisher (https://www.thermofisher.com/uk/) as described below and were FAM-labelled for TAQMan qPCR methodology:

Microtubule associated protein 1 light chain 3 alpha	MAP1LC3A	**Hs01076567_g1**
Heat shock protein family A (Hsp 70) member 1A	HSPA1A	**Hs00359163_s1**
Beta actin	ACTB	**Hs01060665_g1**

Cells were seeded at a density of 100,000 in a 6-well plate and left to attach overnight before treatment with 0.9 nM RuS12·AuNP for 4, 8, 16, 24, 48 and 72 hours. At the end of each time point, cells were washed three times with PBS, trypsinised for 5 minutes and centrifuged for 5 minutes at 1500 g. Cell pellet were stored in 500 µL RNAlater until required. RNA extraction was done using an RNA extraction kit (Qiagen) according to the manufacturer instructions. Briefly, cell pellets were thawed on ice and centrifuged to remove RNAlater and to obtain cell pellets. Next pellets were lysed with 350 µL of buffer RLT and 350 µL of 70% ethanol (dissolved in RNAase-free water), and the solution was pipetted up and down to lyse the cells. The solution 700 µL of was transferred to a spin column in a 2 mL collection tube and centrifuged for 8,000 g on a bench-top centrifuge for 1 minute followed by the addition of 700 µL of buffer RW1 to the spin column (flow through was discarded) and further centrifuged for 1 minute at 8,000 g (flow through discarded). Buffer RBE of 500 µL of was added to spin column and centrifuged for 2 minutes and flow through discarded. Spin column was placed in a 1.5 mL collection tube (RNAase-free water) and RNA was eluted using 40 µL of RNAase-free water added directly to the column, allowed to stand for 1 minute and centrifuged for 1 minute. Concentration and purity of extracted RNA was determined by Nanodrop ND1000 spectrophotometer and samples stored at -80°C until needed. cDNA was synthesised from extracted RNA using a Tetro cDNA synthesis kit (Bioline). All solutions used were briefly vortexed and all preparation were done on ice. Extracted RNA of 500 ng was added to the premix solution. The Premix was made by the addition of 1 µL of oligo (dT) primer, 1 µL of 10 mM dNTP mix, 4 µL 5X RT buffer, 1 µL RiboSafe RNAse inhibitor and 1 µL of Tetro Reverse Transcriptase. Premix solution containing RNA was mixed gently by pipetting. The solution was then incubated at 45°C for 30 minutes and the reaction terminated by incubating at 85°C for 5 minutes. Samples were then chilled on ice before storing at -20°C until needed. RT-PCR was done using 250 ng of cDNA, 1 µL TaqMan® Gene Expression Assay primer (Applied Biosystems,) and 10 µL of TaqMan® Fast Advanced Master Mix (Applied Biosystems) made up to 20 µL with RNAse-free water. The RT -PCR run, consisted of one cycle (polymerase activation) at 95°C for 20 seconds, then 40 cycles of denaturing at 95°C for 1 second and annealing at 60°C for 20 seconds. The data was collected at the annealing stage. β-actin was chosen as the housekeeping genes based on result of western blot. All experiments were done using three biological

replicates for each time point and included three technical replicates. qPCR data was analysed using the ΔΔCt method[1].

References

1. Pfaffl, M. W., A new mathematical model for relative quantification in real-time RT-PCR. *Nucleic Acids Res.* **2001,** *29* (9).
